# In silico analysis of koranimine, a cyclic imine compound from *Peribacillus frigoritolerans* reveals potential nematicidal activity

**DOI:** 10.1038/s41598-022-20461-8

**Published:** 2022-11-07

**Authors:** Jake Adolf V. Montecillo, Hanhong Bae

**Affiliations:** Department of Biotechnology, Yeungnam University, Gyeongsan, Gyeongbuk 38541 Republic of Korea

**Keywords:** Bacteriology, Biomaterials

## Abstract

Pine wilt disease (PWD) is a destructive vector-borne forest disease caused by the nematode *Bursaphelenchus xylophilus*. To date, several options are available for the management of pine wilt disease; however constant development and search for natural products with potential nematicidal activity are imperative to diversify management options and to cope with the possible future emergence of resistance in parasitic nematodes. Here, a combined metabolomics and genomics approach was employed to investigate the chemical repertoire and biosynthetic potential of the bacterial endophyte *Peribacillus frigoritolerans* BE93, previously characterized to exhibit nematicidal activity against *B. xylophilus.* Feature-based molecular networking revealed the presence of diverse secondary metabolites. A cyclic imine heptapeptide, koranimine, was found to be among the most abundant secondary metabolites produced. Genome mining displayed the presence of several putative biosynthetic gene clusters (BGCs), including a dedicated non-ribosomal peptide synthase (NRPS) BGC for koranimine. Given the non-ribosomal peptide nature of koranimine, in silico molecular docking analysis was conducted to investigate its potential nematicidal activity against the target receptor ivermectin-sensitive invertebrate α glutamate-gated chloride channel (GluCl). Results revealed the binding of koranimine at the allosteric site of the channel—the ivermectin binding site. Moreover, the ligand-receptor interactions observed were mostly shared between koranimine and ivermectin when bound to the α GluCl receptor thus, suggesting a possibly shared mechanism of potential nematicidal activity. This study highlights the efficiency of combined metabolomics and genomics approach in the identification of candidate compounds.

## Introduction

Pine wood nematode (PWN) *Bursaphelenchus xylophilus* is an invasive endoparasitic nematode which has been long considered a serious threat to trees of the genus *Pinus*, causing a destructive vector-borne pine wilt disease (PWD)^1^. The occurrence of the disease was first reported in Japan in the 1900s, but it was not until 1972 that the causative nematode was identified^2^. The infection cycle of PWD is facilitated by sawyer beetles *Monochamus* spp. vectors. These vectors emerge from the dead pine trees and migrate to healthy trees to feed on succulent branches bringing along the parasitic nematode. The feeding wounds left by the vectors provide an entry points for the nematode to commence the infection process^3^. The nematodes then migrate to the resin canals where they feed and reproduce. As a result, the functionality of the vascular system is compromised which will eventually lead to the wilting of the tree^4^. The nematode *B*. *xylophilus* is native to North America and believed to have been introduced to Japan at the start of the twentieth century through anthropogenic activities^5^. Since then, the nematode has been devastating pine forests in Japan, spreading to other Asian countries including Korea, China, Taiwan, and even countries in Europe like Portugal and Spain^6–8^ and thus, has become an economically important pest across the globe^9^.

To date, several options are available for the management of PWD. These measures may either be directed against the vector or directly against the nematode, or a combination of both. These can be achieved through aerial spraying of insecticides, fumigation, and trunk-injection of nematicidal compounds (avermectin, abamectin, emamectin benzoate, mesulfenfos, morantel tartrate, and levamisole chloride)^10^. Silvicultural control is also employed which involves clear-cutting and manual removal of infected pine trees^11^. However this physical preventive method requires elimination of pine trees within a radius of 10–50 m surrounding the infected tree which could cause possible loss of uninfected pine trees. Although the use of chemical-based management approaches such as insecticides and nematicidal compounds have been successful in controlling the prevalence of PWD, cautions must be taken into consideration. The use of insecticides has been associated with environmental pollution and may exert adverse impacts on non-target organisms and even on human health^12^. Frequent use of nematicidal compounds on the other hand, may increase the risk of developing resistance against the compound, a phenomenon observed in other parasitic nematodes^13^. Hence, constant development and search for natural products with potential nematicidal activity are imperative to diversify management options and to cope with the possible emergence of resistance in parasitic nematodes including *B*. *xylophilus*.

Since the discovery of penicillin in the 1940s, microorganisms and their specialized secondary metabolites have been drawing much attention from the scientific community^14^. Secondary metabolites of microbial origin are of great interest for their structural diversity and varied bioactivities. The ubiquitous nature of microorganisms coupled with their vast arsenal of secondary metabolites has made them powerful sources of compounds with biotechnological potentials^15^. To this end, a number of secondary metabolites from bacteria have been shown to possess nematicidal/anthelmintic activity against parasitic nematodes^16–20^. Among the known potent anthelminthic compounds, the macrocyclic lactone avermectins and its analogs (e.g., ivermectin and abamectin) originally produced by *Streptomyces avermitilis*, are widely used in agriculture, veterinary and medical fields due to their broad anthelmintic spectrum^21^. Avermectins target the invertebrate glutamate-gated chloride channel (GluCl), a member of the Cys-loop receptor family of ligand-gated ion channels which includes nicotinic acetylcholine and type 3 5-hydroxytryptamine receptors (nAChRs and 5-HT_3_Rs) and inhibitory γ-aminobutyric acid (GABA) type A and glycine receptors (GABA_A_Rs and GlyRs)^22^. The anthelmintic compound acts as a strong agonist, activating the GluCls present in nerve or muscle cells at nanomolar concentrations causing hyperpolarization that will result to subsequent paralysis^23–25^. Although the microbial secondary metabolites are highly promising agents, it has been believed that only a portion of the organisms’ total metabolites (metabolome) have been tapped so far due to the laborious traditional screening and compound identification approaches^26^.

Secondary metabolites are typically purified, identified and characterized after an extensive screening of crude extracts with biological activities—a so called bioactivity-based screening approach. Though proven to be efficient, still the approach is plagued by high rediscovery rates of known compounds and limited to the detection of the highly abundant metabolites^27^. Recently, with the availability of genome sequences, the application of genome mining approach which tries to identify biosynthetic gene clusters (BGCs) and predict corresponding compound has been widely pursued as a complementary approach to the classical bioactivity-guided screening^28^. Rapid annotation or identification of compounds present in the metabolome prior to purification is also a crucial step, and also been considered a stumbling block in the natural product research arena^29^. In this context, the application of liquid chromatography coupled with tandem mass spectrometry (LC–MS/MS) in untargeted metabolomics studies has allowed the global analysis of secondary metabolites due to the high sensitivity and flexibility of the technique. Besides, the technique also provides important structural information of the detected compounds, aiding in the identification process^30^. In addition, the introduction of molecular networking, an approach that enables the analysis and organization of tandem mass (MS/MS) spectrometry data based on structural similarity has also provided great assistance and improved efficiency in natural product discovery^31^. The Global Natural Products Social Molecular Networking (GNPS), a web-based platform, has enabled the molecular networking algorithm accessible to the scientific community^32^. This online pipeline has been successfully used to identify known compounds, analogs or derivatives, and even the discovery of novel compounds from different complex biological samples^33–38^.

Studying the interactions of natural products with their probable biological targets can render insights into their potential drug applications. Over the past years, computational molecular docking methods have been frequently employed in structure-based drug designing, owing to its ability to detect appropriate binding sites in the targets and the interactions of the small molecule (ligand) with high accuracy. Molecular docking programs work by calculating probable ligand conformations or poses in the target active site and ranking them based on their individual binding energies^39–41^. A number of studies have demonstrated the efficiency of molecular docking approaches in predicting potential drug candidates as validated by their corresponding in vitro studies^42–45^. Hence, this in silico screening can significantly accelerate the identification of potential natural product drug candidates while minimizing experimental labor.

Previous study from our group has identified bacterial endophytes from pine trees showing potent nematicidal activity against *B*. *xylophilus*^46^. One of the bacterial endophytes was identified as *Peribacillus frigoritolerans* BE93^47^, formerly identified as *Bacillus* sp. BE93. Though shown to possess nematicidal activity, the chemical potential of BE93 has not been characterized. In this study, we used a combined metabolomics and genomics approach to investigate the biosynthetic potential and chemical repertoire of BE93. Untargeted LC–MS/MS was used to characterize the metabolome of BE93 grown in different culture media. The MS/MS data were then analyzed using the feature-based molecular networking (FBMN) workflow within the GNPS web platform analysis environment^48^. The genome sequence of BE93 was analyzed using antiSMASH 5.0^49^ and PRISM^50^ to identify specialized BGCs. Additionally, in silico analysis of the identified compound of interest was carried out using AutoDock Vina^51^ within the UCSF Chimera^52^ working space to investigate its potential nematicidal activity.

## Results

### Metabolic profiling of BE93

**Figure 1:**
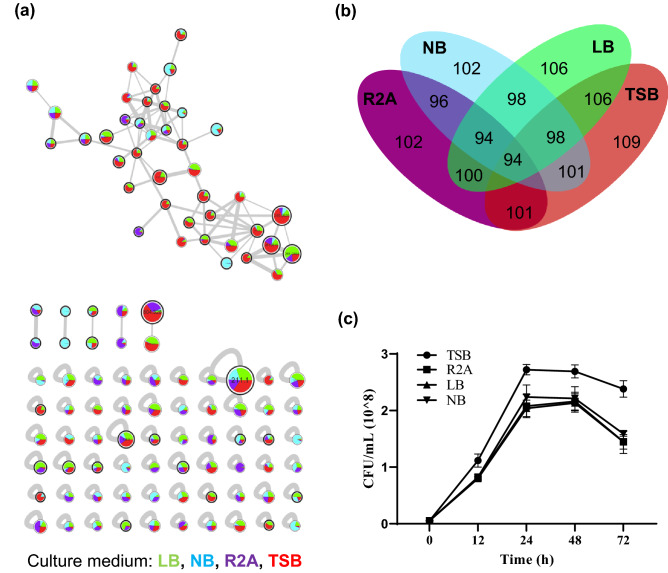
Metabolomics analysis of *Peribacillus frigoritolerans* BE93 grown in different culture media. (**a**) Feature-based molecular networking (FBMN) of the detected secondary metabolites from BE93 culture extracts. Each node represents one fragmentation spectrum from a detected compound, node size represents the summed ion intensity from all samples, edge thickness indicates the relative similarity of the tandem mass (MS/MS) data between nodes, and the pie charts indicate the relative abundance of each compound from the different samples. Nodes having a putative library match are outlined in black. Colors represent the culture medium. (**b**) Venn diagram showing the counts and distributions of nodes among the different culture media. (**c**) Growth curve of BE93 in different culture media.

To characterize the secondary metabolites produced by BE93, four different media [tryptic soy broth (TSB), R2A, nutrient broth (NB), and Luria-Bertani (LB)] were used for the cultivation of the bacterium. The culture extracts were then subjected to LC-MS/MS analysis and the data were processed for the establishment of molecular network using the GNPS online platform. After background and media subtraction, molecular networks were established with a total of 110 compounds or nodes (Fig. 1a). Majority of these compounds were detected and shared in all four culture media conditions, although distinct compounds were also found to be media specific, and with TSB having the most number of detected compounds (Fig. 1b). In addition, the abundance of the detected compounds varied from one culture medium to another. Some of the detected compounds were successfully annotated while the other compounds remained unknown. Of the four culture media used, TSB was the most favorable medium for the cultivation of the bacterium (Fig. 1c).

### Structural validation and characterization of koranimine production

**Figure 2:**
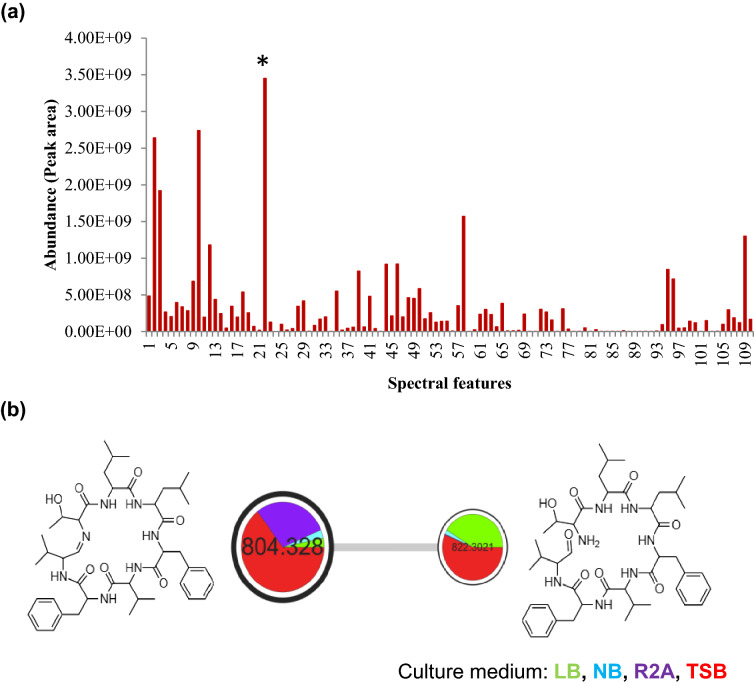

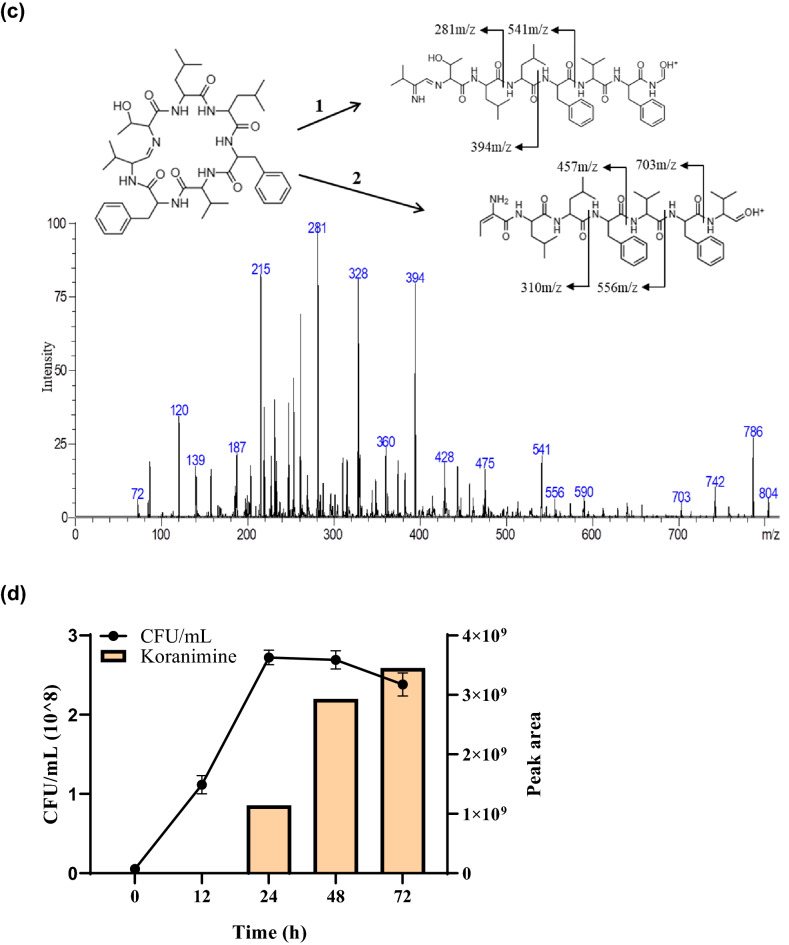
Characterization of koranimine from *Peribacillus frigoritolerans* BE93. (**a**) Relative abundance of the detected spectral features in tryptic soy broth (TSB) medium. Asterisk (*) represents koranimine. (**b**) Koranimine molecular network showing the structure of koranimine and its open aldehyde form. Pie chart represents relative abundance in each culture medium. (**c**) Elucidated tandem mass (MS/MS) fragmentation pathways of koranimine. (**d**) Time-dependent production of koranimine in TSB medium.

In our previous study, the culture extract of BE93 grown in TSB showed nematicidal activity against *B*. *xylophilus*^46^. Considering the previous result, we then next closely characterized the secondary metabolites detected in the culture extract of BE93 cultivated in TSB medium. Out of the total 110 compounds or spectral features, 109 were detected in the TSB culture extract, each with varying abundance (Fig. 2a). Spectral feature 22 with molecular ion of 804.328 m/z was found to be the most abundant spectral feature. Other abundant spectral features were also observed with molecular ions ranging from 211.10 to 261.05 m/z, representing compounds of the same class (Supplementary Table S1, Fig. S1). The molecular ion 804.328 m/z [M+H] was annotated by the GNPS spectral library as a close match to koranimine, a cyclic imine compound isolated from *Bacillus* sp. NK2003^53^ (Supplementary Fig. S2). A molecular network of koranimine was also observed, with a single node having a molecular ion of 822.302 m/z [M+H] connected to koranimine (Fig. 2b). The 822.302 m/z [M+H] molecular ion represented the open form of koranimine, the direct result of reductive release from the biosynthetic assembly. Moreover, koranimine and its open form were observed to be produced in all culture media used in varying degree of abundance. We further verified the identity of the detected koranimine in our sample by manually assessing the fragment spectrum (MS/MS) of the compound. Two fragmentation pathways were elucidated (Fig. 2c). Ring opening in pathway 1 occurred through cleavage between the sixth and seventh amino acids (Phe-Val). Successive cleavage between residues resulted in the generation of different observed fragment ions. The cleavage between the seventh and first amino acids (Val-Thr) resulted in the opening of the ring as indicated in pathway 2. We also evaluated the production of koranimine in the TSB medium (Fig. 2d). BE93 showed a time-dependent production of koranimine, commencing at the late exponential phase and gradually increased towards the stationary phase. From these results, we have speculated that koranimine, a dominant secondary metabolite, might be responsible for the reported nematicidal activity of BE93.

### Whole genome sequencing and identification of biosynthetic gene clusters

**Figure 3:**
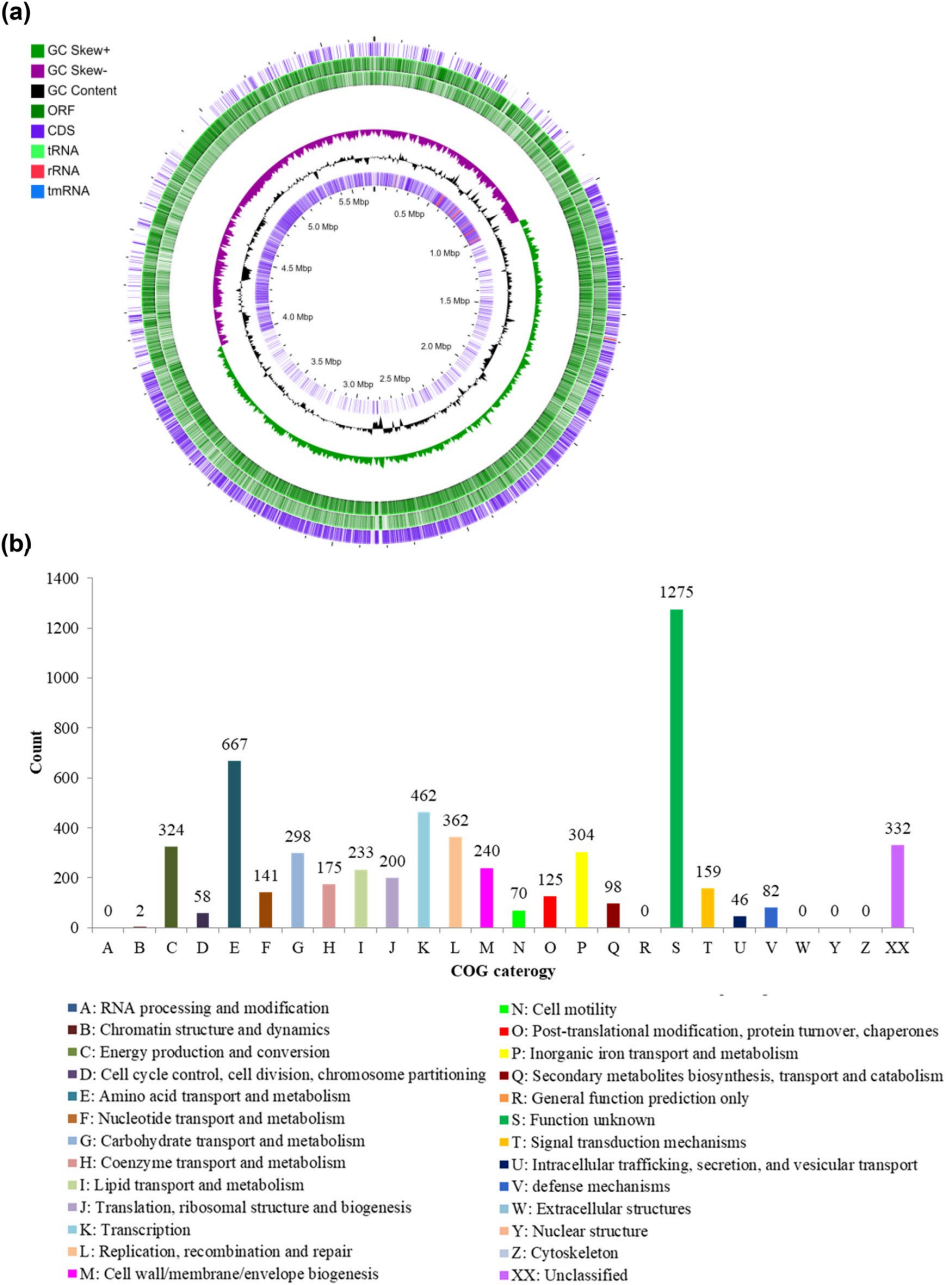

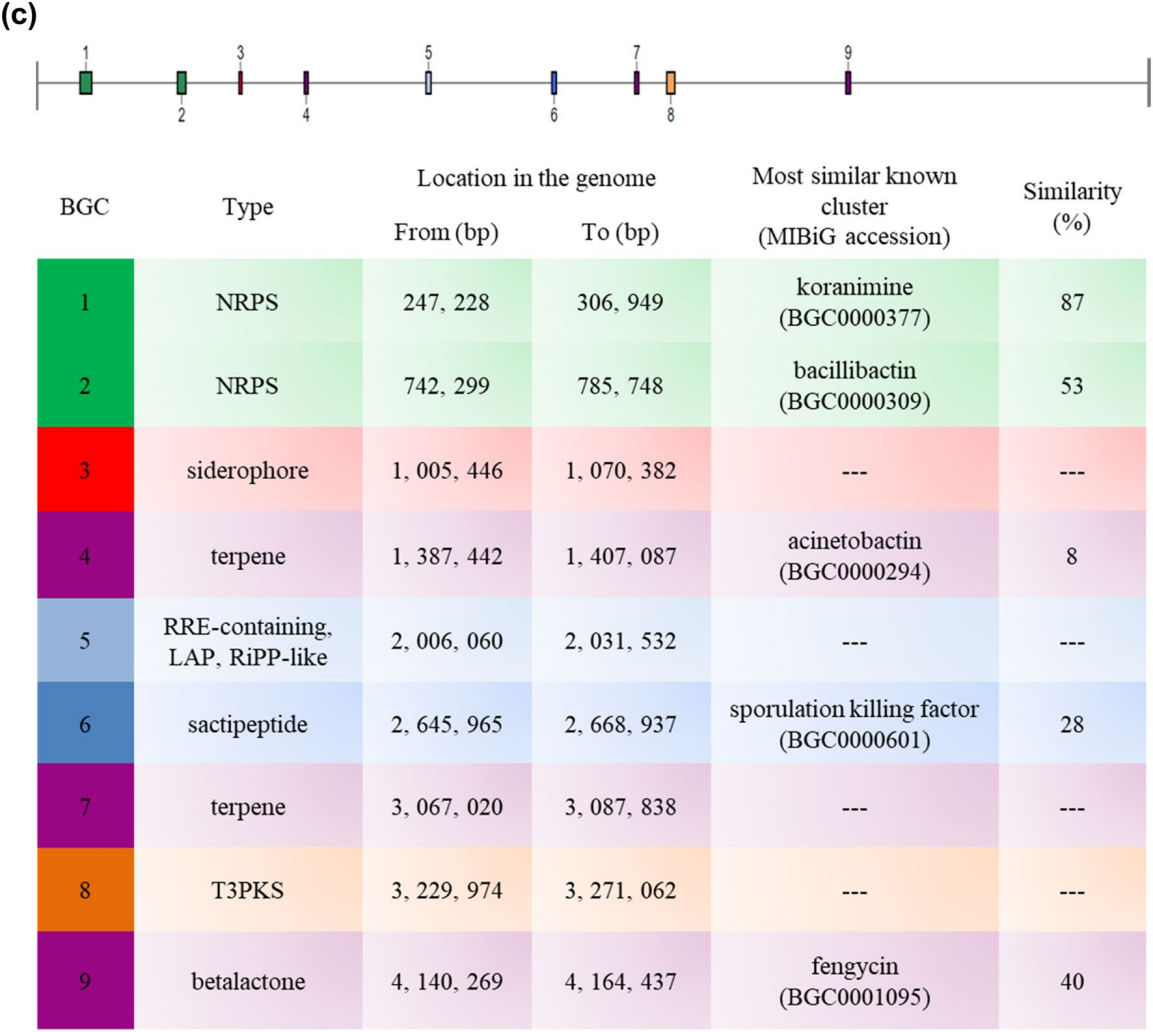
Analysis of the whole genome sequence and biosynthetic gene clusters (BGCs) of *Peribacillus frigoritolerans* BE93. (**a**) Circular whole genome map with marked characteristics. (**b**) Clusters of orthologous group (COG) classification of coding sequences (CDS) based on eggNOG annotation. (**c**) Putative BGCs predicted using the antiSMASH 5.0 algorithms.

Whole genome sequencing was performed to examine the biosynthetic potential of BE93 and to further verify the production of koranimine, through the identification of BGCs encoded in the genome. The bacterium has a genome size of 5, 693, 946 base pair (bp) with 40.54 mol % G + C content, and 5, 653 identified coding sequences (Fig. 3a). Putative functions of the coding sequences were predicted using the eggNOG platform (Fig. 3b). Out of the 5, 653 coding sequences 5, 321 were successfully assigned to a specific clusters of orthologous group (COG) category, accounting for 94% of the total coding sequences. The remaining 332 coding sequences were unclassified.

The antiSMASH 5.0 algorithms were used to analyze the genome sequence for the presence of BGCs. The analysis revealed 9 putative BGCs comprising 2 non-ribosomal peptide synthases (NRPS), 2 terpenes, 1 siderophore, post-translationally modified peptides (RiPPs), sactipeptide, type III polyketide synthase (T3PKS), and betalactone (Fig. 3c). One of the predicted BGCs showed high similarity (> 80%) to the known koranimine BGC in the MIBiG database, substantiating the detected koranimine production in the bacterium. Three of the other predicted BGCs displayed moderate levels of similarity (28–53%) to known BGCs, while the remaining 5 BGCs exhibited low to no similarity. The consolidated length of the predicted BGCs was 272,302 bp, accounting for around 4.8% of the BE93 genome.

### Characterization of koranimine biosynthetic gene cluster

**Figure 4:**
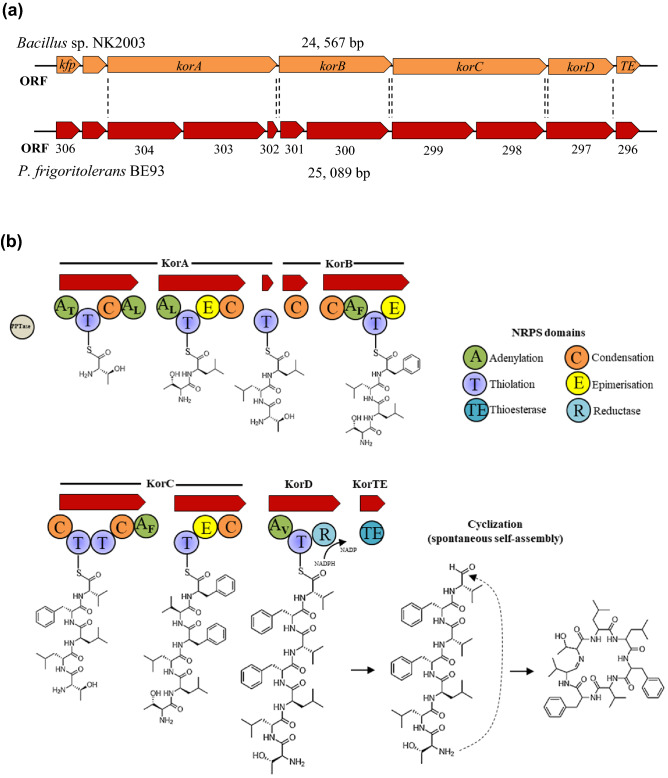
Characterization of koranimine biosynthetic gene cluster. (**a**) Comparison of koranimine biosynthetic gene cluster (BGC) gene organization detected in *Peribacillus frigoritolerans* BE93 with that of *Bacillus* sp. NK2003. (**b**) Non-ribosomal peptide synthase (NRPS) domains and modules found in the BE93 koranimine BGC and the proposed pathway for the biosynthesis of koranimine.

Koranimine and its biosynthesis genes were first characterized in the soil dwelling bacterium *Bacillus* sp. NK2003^53^. The BGC for koranimine in BE93 shared 87% similarity with the koranimine BGC of *Bacillus* sp. NK2003. The koranimine BGC of *Bacillus* sp. NK2003 is composed of 6 open reading frames (ORFs) representing the core biosynthesis genes encoding phosphopantetheinyl transferase (*kfp*), koranimine (*korA*, *korB*, *korC*, *korD*), type II thioesterase (*korTE*), and one hypothetical gene. Close analysis of the BE93 koranimine BGC revealed striking different gene architecture (Fig. 4a). Unlike the previously characterized BGC, 11 ORFs were present in the koranimine BGC of BE93 stretching 25,089 bp. Three ORFs were found to comprise *korA*, and 2 ORFs for *korB* and *korC*. Amino acid identity of the individual ORFs displayed high level of similarity to the core biosynthesis genes of koranimine BGC (Table 1).

**Table 1:** Open reading frames (ORFs) representing the koranimine biosynthetic gene clusters (BGCs) in *Peribacillus frigoritolerans* BE93.

*Bacillus* sp. NK2003			BE93			Function	Identity (%)
ORF^a^	Base pair	Amino acids	ORF	Base pair	Amino acids		
*kfp*	714	237	306	711	236	Phosphopantetheinyl transferase	87
*korA*	7815	2605	304	4002	1333	NRPS	89
			303	3516	1171	NRPS	89
			302	267	88	NRPS	90
*korB*	4710	1545	301	540	179	NRPS	93
			300	4158	1385	NRPS	92
*korC*	7707	2568	299	4197	1398	NRPS	89
			298	3441	1146	NRPS	90
*korD*	2889	962	297	2904	967	NRPS	92
*korTE*	732	243	296	693	230	thioesterase	88

^a^Genes encoding phosphopantetheinyl transferase (*kfp*), koranimine (*korA*, *korB*, *korC*, *korD*), type II thioesterase (*korTE*), non-ribosomal peptide synthase (NRPS).

The difference in the gene architecture of the koranimine BGC between BE93 and *Bacillus* sp. NK2003 was also evident in the biosynthesis NRPS domains and modules. Previous report revealed 5 adenylation domains of the koranimine BGC, lacking a dedicated adenylation domain for 1 leucine residue. Here, the koranimine BGC of BE93 displayed 6 adenylation domains, including a dedicated adenlylation domain for installing the second leucine residue. As expected, the groupings of the NRPS modules were also different as the core genes were divided into several ORFs. Figure 4b shows the NRPS domain architecture of koranimine BGC and the proposed biosynthesis pathway of koranimine in BE93.

### Comparative in silico analysis of koranimine and ivermectin

**Figure 5:**
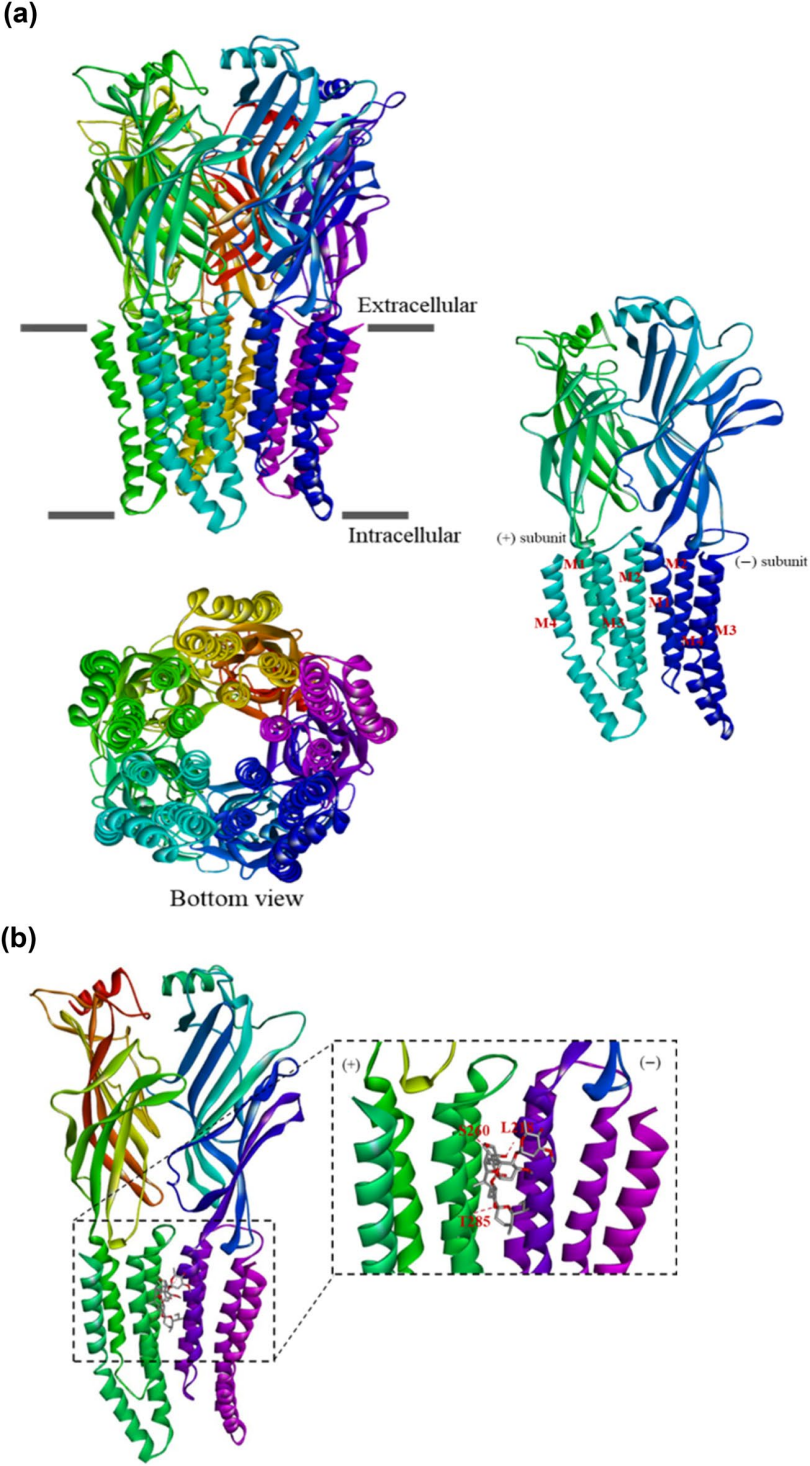

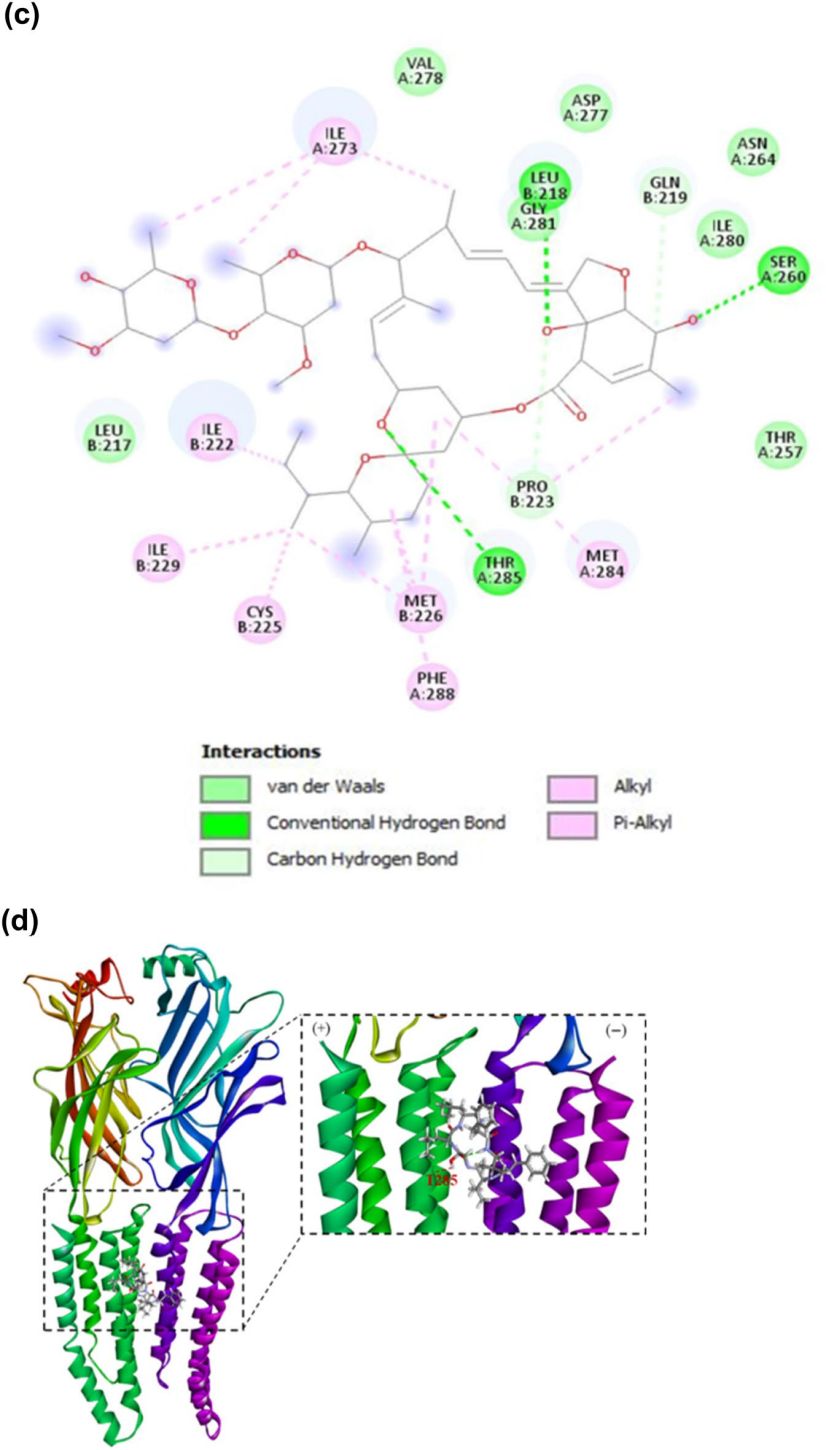

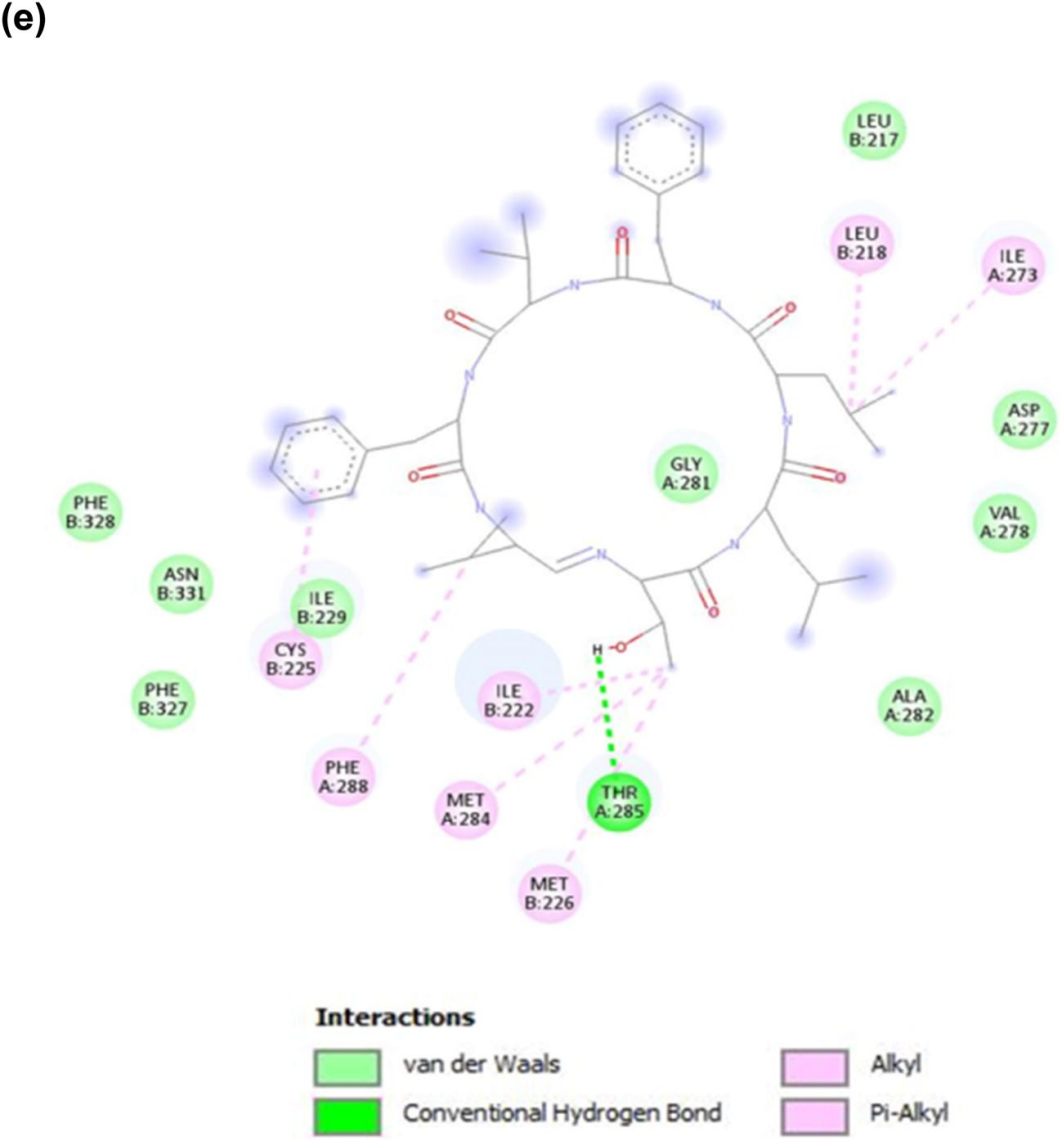
Molecular docking simulation of koranimine and ivermectin with *Caenorhabditis elegans* α GluCl receptor. (**a**) Three-dimensional (3D) protein model of *C. elegans* ivermectin-sensitive invertebrate α glutamate-gated chloride channel (GluCl) obtained from research collaboratory for structural bioinformatics protein data bank (RCSB PDB). (**b**) Best docking pose of ivermectin bound to the allosteric site of α GluCl receptor. (**c**). Ligand-receptor interactions between ivermectin- α GluCl receptor shown in 2D. (**d**) Best docking pose of koranimine bound to the allosteric site of α GluCl receptor. (**e**) Ligand-receptor interactions between koranimine- α GluCl shown in 2D.

To assess the potential of koranimine as a nematicidal compound, in silico analysis through molecular docking was performed. We chose the ivermectin-sensitive invertebrate glutamate-gated chloride channel (GluCl) (Fig. 5a) as the target protein or receptor, considering the comparable molecular weight (804 g/mol) and the macrocyclic nature of koranimine to the known macrocyclic nematicidal compound ivermectin (875 g/mol). We first conducted a re-docking experiment of *Caenorhabditis elegans* α GluCl receptor^54^ with its cognate agonist ivermectin to demonstrate the accuracy of our adopted molecular docking system. The re-docking experiment resulted in a good docking score with a binding energy of − 10.3 kcal/mol. Close analysis of the best docking pose of ivermectin revealed highly congruent ligand-receptor conformation with the previous report^54^. The docking results displayed the binding of ivermectin at the subunit interfaces of the transmembrane domains, the allosteric site proximal to the extracellular side (Fig. 5b,c). Specifically, ivermectin showed binding between the M3 α-helix of the principal (+) subunit and the M1 α-helix of the adjacent complementary (‒) subunit, and with the benzofuran structure contacting the M2 pore-lining α-helix of the (+) subunit. Analysis of the ligand-receptor interactions revealed three hydrogen bonds formed between the ligand and the M2(+) Ser260, M3(+) Thr285, and M1(‒) Leu218 residues of the receptor. Moreover, extensive van der Waals and hydrophobic interactions were also observed such as alkyl, and pi-alkyl interactions.

Using the *C. elegans* α GluCl receptor, we also performed docking experiment with koranimine. Having shown the ligand-receptor interactions of ivermectin—α GluCl receptor, a good comparative evaluation can be employed. The docking results showed the binding of koranimine at the subunit interfaces of the transmembrane domains of the α GluCl receptor similar to the one observed in ivermectin, with a binding energy of − 8.66 kcal/mol (Fig. 5d,e). Koranimine positioned between the M3(+) α-helix subunit and the M1(‒) α-helix subunit of α GluCl receptor. A close look on the formed ligand-receptor interactions displayed a hydrogen bond with the M3(+) Thr285 residue of the receptor. Extensive van der Waals and hydrophobic interactions, mostly found in ivermectin—α GluCl receptor interaction were also observed, especially with the amino acid residues deemed important for ivermectin binding [M1(‒) Leu218 and M3(+) Gly281].

**Figure 6:**
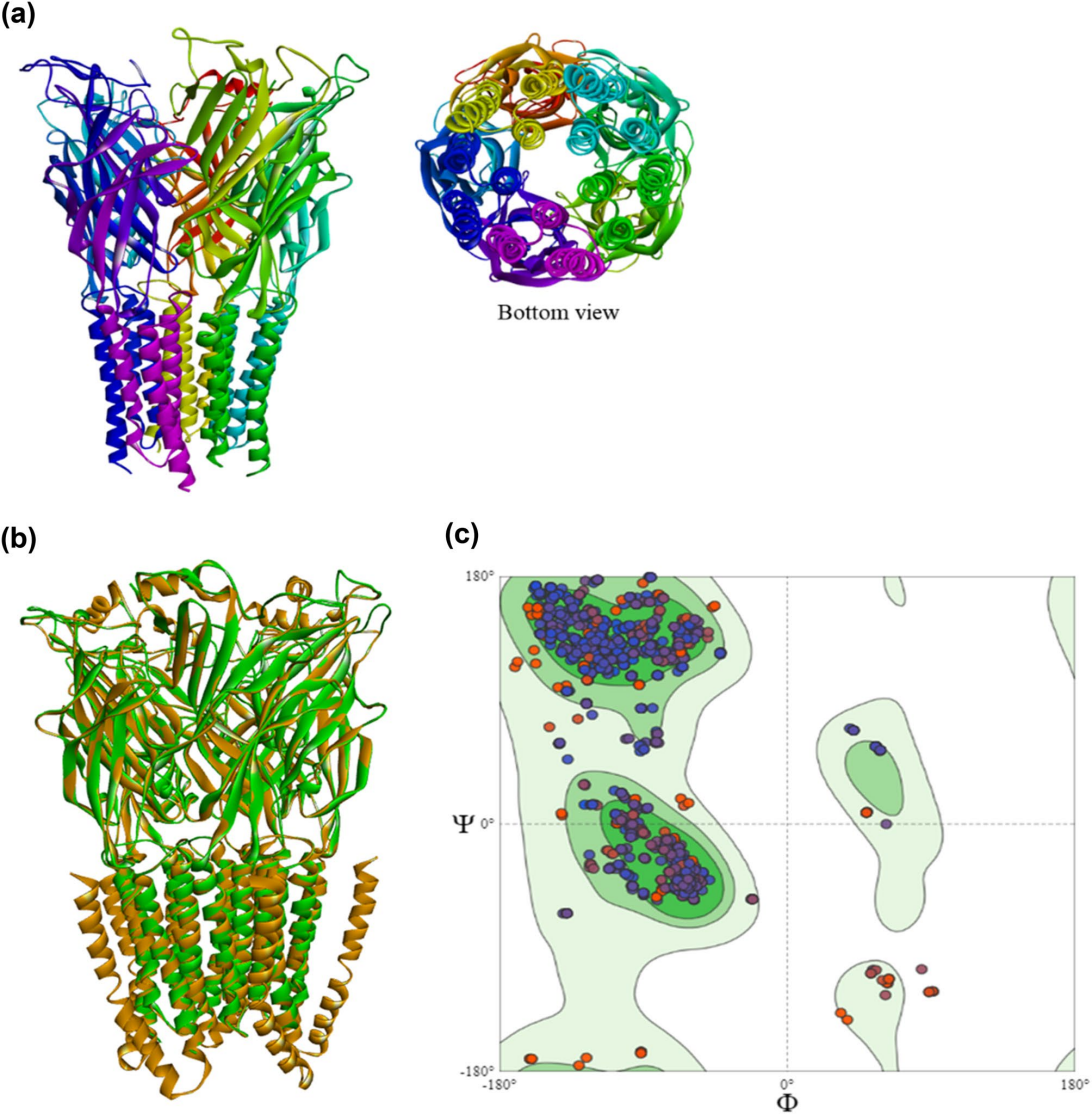
Homology modelling of *Bursaphelenchus xylophilus* α glutamate-gated chloride channel (GluCl) receptor. (**a**) Three-dimensional (3D) model of *B*. *xylophilus* α GluCl receptor obtained through homology modelling. (**b**) Superimposed 3D structures of α GluCl receptor from *B*. *xylophilus* (green) and *C*. *elegans* (brown). (**c**) Ramachandran plot showing the quality assessment of *B*. *xylophilus* α GluCl receptor 3D model.

Taken into account the results from the comparative ligand-receptor interactions of ivermectin and koranimine with *C*. *elegans* α GluCl receptor, we then sought to investigate the compounds’ interactions with the α GluCl receptor of the target nematode *B*. *xylophilus.* The α GluCl receptor of *B*. *xylophilus* has not been resolved yet through crystallographic methods. However, the gene encoding for the receptor is present in the genome of *B*. *xylophilus.* In this regard, we used homology modelling to obtain the 3-dimensional (3D) structure of the α GluCl receptor from *B*. *xylophilus.* The identified α GluCl receptor of *B*. *xylophilus* (CAD5232901) displayed 72.40% protein sequence similarity with the *C*. *elegans* α GluCl receptor (3RHW_A). The *C*. *elegans* α GluCl receptor was then used as a template for the homology modelling. The built 3D model of the α GluCl receptor is shown in Fig. 6a,b. The Ramachandran plot of the modelled structure showed decent quality assessment, exhibiting more number of residues in the most favorable regions (96.04%), and low number of residues were found in the disallowed regions (Fig. 6c). The ERRAT plot also showed good resolution of the modelled 3D structure (Supplementary Fig. S3). The modelled structure was then used for the molecular docking experiment.

**Figure 7:**
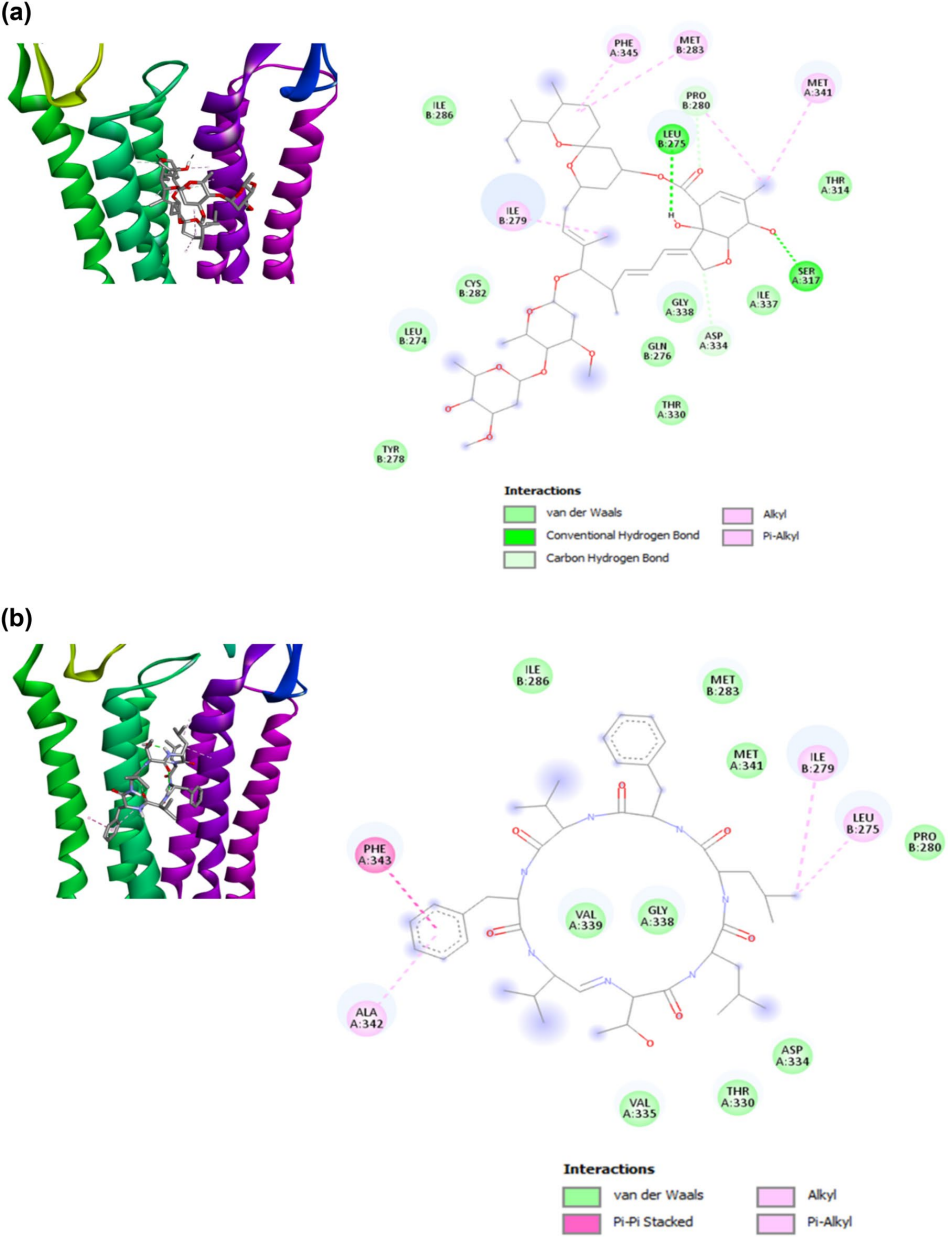
Molecular docking simulation of koranimine and ivermectin with *Bursaphelenchus xylophilus* α glutamate-gated chloride channel (GluCl) receptor. (**a**) Best docking pose of ivermectin bound to the allosteric site of α GluCl receptor (left) and the ligand-receptor interactions shown in 2D (right). (**b**) Best docking pose of koranimine bound to the allosteric site of α GluCl receptor (left) and the ligand-receptor interactions shown in 2D (right).

Results from the molecular docking of ivermectin and α GluCl receptor of *B*. *xylophilus* revealed similar binding pose with that of ivermectin—*C*. *elegans* α GluCl receptor interaction (Fig. 7a), with ivermectin positioned between the M3(+) α-helix and the M1(‒) α-helix subunits of receptor having a binding energy of − 8.7 kcal/mol. Two hydrogen bonds were observed, formed between the ligand and the M2(+) Ser317 [counterpart of M2(+) Ser260 of *C*. *elegans* α GluCl receptor] and the M1(‒) Leu275 [M1(‒) Leu218 equivalent] residues of the receptor. Hydrophobic interaction (van der Waals) was only observed between the ligand and the M3(+) Thr330 [M3(+) Thr285 equivalent] residue of the receptor, unlike with the hydrogen bond observed in the ivermectin—*C*. *elegans* α GluCl receptor interaction. Extensive interactions such as van der Waals, and hydrophobic alkyl, and pi-alkyl interactions were also observed. On the other hand, the best docking result of koranimine with the *B*. *xylophilus* α GluCl receptor still positioned the ligand in the allosteric site, between the two subunits M3(+) and M1(‒) of the receptor with a binding energy of − 7.0 kcal/mol (Fig. 7b). Koranimine formed van der Waals and hydrophobic interactions with the important key residues of the receptor, M1(‒) Leu275, M3( +) Thr330, M3( +) Gly330, and with some other residues observed in the ivermectin—*B*. *xylophilus* α GluCl receptor interaction. Taken together, these results reveal the potential of koranimine as a nematicidal compound that targets the invertebrate α GluCl channel.

## Discussion

Secondary metabolites, also known as natural products are small molecules produced by a diverse group of living organisms. Bacterial endophytes, in particular are long been known sources of structurally diverse bioactive compounds with potential biotechnological applications. In this study, we explored the secondary metabolites of BE93 to uncover potential compound responsible for the observed nematicidal activity of the bacterium against *B*. *xylophilus*. We employed a combination of metabolomics and genomics approach to reveal the chemical and biosynthetic potential of BE93. In addition, in silico analysis through molecular docking was also employed to evaluate the nematicidal potential of the compound of interest.

The metabolic profile of BE93 grown in different culture media displayed a wide array of secondary metabolites. These secondary metabolites were mostly detected and shared in all culture media used, with varying abundance. Moreover, the GNPS workflow has allowed the reliable annotation of certain compounds from the culture extracts. A compound of interest, koranimine (804.328 m/z [M+H]) was shown to be present in all media used, and found to be the most abundant compound produced in the TSB medium. MS/MS has enabled the use of product ions for the identification of fragmentation pattern useful for the validation of compounds^55^. The identity of the detected koranimine was further validated through manual inspection of the MS/MS spectrum and the determination of the signature fragment ions. The elucidated fragmentation pathways coincided with the reported fragmentation pathways of koranimine^53^, thereby confirming the identity of the detected compound. Koranimine was first described from the environmental isolate *Bacillus* sp. NK2003 as an imine-containing cyclic peptide^53^. The compound is composed of 7 amino acids Thr-Leu-Leu-Phe-Val-Phe-Val and is produced through non-ribosomal biosynthesis. Previous assessment of the bioactivity of koranimine showed that the compound had no inhibitory effects on the growth of pathogenic bacteria and fungi^53^, suggesting that the compound does not act as an antimicrobial agent. At the time of writing this manuscript, no biological activities have been attributed yet to koranimine. Of note, peptides produced through non-ribosomal biosynthesis usually possess various potent bioactivities. Members of this chemical group, aside from antibiotics, include anticancer drugs such as bleomycin^56^, immunosuppressants like cyclosporin^57^, mycotoxins^58^, and broad spectrum insecticidals such as beauvericin^59^. Koranimine as a non-ribosomal peptide may possess bioactivities of relevant interests, such as nematicidal activity of which the producer bacterium BE93 has been shown to exhibit.

The production of secondary metabolites in microorganisms is greatly affected by culture media composition^60^. Here, the observed variation in the abundance of compounds produced in the different culture conditions can be attributed to the differential growth of the bacterium in each medium. BE93 was found to prefer a plant-based rich medium TSB, compared to other rich media like LB and NB, and low nutrient medium R2A for growth and secondary metabolite production. In TSB medium, koranimine production was observed to peak at the start of the stationary growth phase of the bacterium. This time-dependent increase of koranimine production was similarly detected in *Bacillus* sp. NK2003^53^. The production of secondary metabolites in microorganisms mostly commences at late exponential and dominates upon entering the stationary phase^61^.

The genome of BE93 displayed the presence of koranimine BGC, with high similarity to the previously characterized BGC in *Bacillus* sp. NK2003^53^. Other putative BGCs were also observed suggesting the ability of the bacterium to synthesize diverse specialized metabolites. The koranimine BGC of *Bacillus* sp. NK2003 is represented by the core biosynthesis genes *kfp*, *korA*, *korB*, *korC*, *korD, korTE* and one hypothetical gene. Only 5 adenylation domains were characterized as opposed to the expected 7 for the heptapeptide product. Installing the second and third leucine amino acids have been shown to be facilitated by a single leucine adenlytion domain of KorA. A long-range activation of KorC has also been observed, in which the tandem thiolation domains of KorC were both charged with valine amino acid by the adenylation domain of KorD. KorD hence, is responsible for the incorporation of 2 valine amino acids the fifth and the seventh valine residues, respectively. The NRPS module KorD, aside from installing the 2 valine residues, has been shown to be involved in the NADPH-dependent reductive release of the compound, forming a linear product with terminal aldehyde. Spontaneous macrocyclization facilitated by the terminal aldehyde yields the cyclic imine compound^53^. Though all the koranimine core biosynthetic genes were found in BE93, the BGC showed a pronounced difference in the gene organization and NRPS domains. Strikingly, the koranimine BGC of BE93 was found to contain 6 adenylation domains, including a third dedicated adenlylation domain in KorA module responsible for installing the second leucine residue. Though different in their NRPS domain organization, both the *Bacillus* sp. NK2003 and BE93 shared the unusual aspects of the koranimine biosynthesis.

The culture extract of BE93 grown in TSB has been shown to have potent nematicidal activity against different developmental stages of *B*. *xylophilus*^46^. Taken into account this previous finding, coupled with the dominant presence of koranimine in the TSB medium, an in silico analysis through molecular docking was performed to examine the nematicidal potential of the compound. Using the α GluCl channel of both *C*. *elegans* and *B*. *xylophilus* as the target receptor, koranimine displayed binding specificity at the allosteric site known for ivermectin binding^54^. Moreover, extensive van der Waals and hydrophobic interactions formed between koranimine and the receptor were observed, specifically with those residues reported to have been involved in ivermectin binding: M1(‒) residues Leu217, Leu218, Ile222, Met226, Pro223, and M2( +) residues Asp227, Gly281, Met284, Thr285, Phe288^22^. Counterparts of these amino acid residues were mostly observed as well in the *B*. *xylophilus* α GluCl-koranimine interaction. Generally, van der Waals and hydrophobic interactions allow the ligand to achieve stable conformation resulting in a stable receptor-ligand complex, hence rendering better activity of the ligand^62^. Ivermectin acts by activating the α GluCl channels present in nerve or muscle cells causing hyperpolarization that will result to subsequent paralysis^23–25^. This activation is the result of the conformational change of the channel upon binding of ivermectin to the allosteric site^54^. The binding of koranimine to the allosteric site of α GluCl channel and the extensive ligand-receptor complex interactions shared between koranimine and ivermectin greatly suggest the strong affinity of koranimine to the target Cys-loop receptor α GluCl. These further suggest a potential nematicidal activity, similar to that displayed by ivermectin.

On the other hand, koranimine is a macrocyclic imine-containing compound, a member of known natural products that contain a carbon–nitrogen double bond in the structure like nostocyclopeptide^63^, scytonemide A^64^, and lugdunin^65^. Other members of this group are marine biotoxins produced by dinoflagellates such as spirolides, gymnodimines, pinnatoxins, and among others. These toxins target the nicotinic acetylcholine receptors (nAChRs) which are cholinergic pentameric receptors that formed ligand-gated ion channels in neurons and postsynaptic side of the neuromuscular junction. Interaction of the toxin with the receptor results in a paralysis. In these toxins, it has been deduced that the imine functional group serves as the common pharmacophore as it showed important role in tethering the toxins to their binding site^66^. It can be noted that nACh and GluCl receptors are members of Cys-loop family of receptors, of which ivermectins have shown to have activities^22^. Hence, the imine functional group of koranimine which is considered a pharmacophore in marine biotoxins, further substantiates the affinity of the compound towards Cys-loop receptors and its potential nematicidal activity. Single compound isolation, characterization and in vitro studies of the compound to further probe its nematicidal activity are currently underway.

Thus far, there have been no reports of ivermectin resistance in *B*. *xylophilus,* however, the possibility of developing resistance remains in place, as observed in other parasitic nematodes^13^. The mechanism of ivermectin resistance in some parasitic nematodes is far from clear, although most studies have suggested the involvement of ATP-binding cassette (ABC) transporters such as P-glycoproteins^67,68^. Ivermectin has been shown to be an excellent substrate of some ABC transporters. The affinity of ivermectin to these transporters renders its reduced intracellular concentration, subsequently resulting to reduced ivermectin sensitivity in nematodes^68^. It is then apparent that the observed ivermectin resistance in some nematodes did not arise from the modification of the ivermectin compound or alteration of the ivermectin targets. Hence, a compound with potentially the same mechanism of action with ivermectin, like koranimine can still be seen as a promising nematicidal agent in managing ivermectin resistant nematodes as long as the compound is not subject to efflux by the ABC transporters.

In general, this study revealed the production of a cyclic imine compound koranimine with potential nematicidal activity from a bacterial endophyte BE93. The compound can be seen as a promising nematicidal agent and could diversify treatment options for the management of PWD caused by *B*. *xylophilus.* Nonetheless, in vitro study of the pure compound must be conducted to further confirm the in silico result of this study. On the other hand, this study also highlighted the efficiency of the combined metabolomics and genomics approach in the identification of candidate compounds.

## Methods

### Bacterial strain and culture conditions

*Peribacillus frigoritolerans* BE93 was obtained from our in-house collection of bacterial endophytes isolated from 4 pine tree species in Republic of Korea^46^. The strain was routinely cultured in Bacto tryptic soy broth (TSB) at 30 °C with shaking (150 rpm) for 24 h to generate the seed culture. For secondary metabolite analysis, a 1:100 dilution of the seed culture was inoculated into Difco Nutrient Broth (NB), Difco Luria-Bertani (LB), MBCell R2A Broth, and TSB. The cultures were then incubated at 30 °C, 150 rpm for 3 days. Bacterial growth was also determined every 12 h through standard plate dilution technique. 

### Secondary metabolite extraction and LC–MS/MS analysis

To extract the secondary metabolites of BE93, the culture broth from each media was mixed with an equal volume of ethyl acetate followed by sonication for 30 min. The organic phase was then separated from the medium and concentrated using a rotary evaporator. The concentrated extract was then dissolved in 1 mL of HPLC-grade methanol and filtered with a syringe filter (0.45 μm).

The ethyl acetate extracts were then subjected to liquid chromatography- mass spectrometry (LC–MS) analysis using LCMS-8040 tandem quadruple mass spectrometer (Shimadzu, Tokyo, Japan). Chromatographic method consisted of solvent A (0.1% formic acid in HPLC-grade water) and B (100% acetonitrile). The flow rate was set to 0.4 mL/min in a C18 column (150 × 4.6 mm, 2.5 μm; Advanced Chromatography Technologies) under the following conditions: 0–1 min, 5% B; 1–30 min, 40% B; 30–52 min 100% B; followed by hold of 100% B for 4 min; and column reconditioning with 5% B for 4 min. The mass spectrometer was operated in positive mode data dependent MS/MS scans, monitoring a mass range from 50 to 2000 atomic mass units (amu), capillary voltage of 3500 V, nebulizer at 60 psi, dry gas flow 15 L/min, and dry temperature at 320 °C.

### Feature-based molecular networking

The FBMN workflow of the GNPS web platform was used to generate the molecular networks as it allows for the discrimination of isomers, redundant fragment ions, and quantitative evaluation of the molecular network^48^. The raw LC-MS/MS data files were converted into mzXML file format using the Proteowizard MSConvert^69^ and subsequently processed using MZmine 2^70^ to generate the MS/MS mascot generic format (MGF) and quantification comma-separated values (CSVs) files required for the FBMN workflow. All the files for the FBMN workflow are available through the mass spectrometry interactive virtual environment (MassIVE) data repository under the accession code MSV000088477.

For the FBMN workflow, default settings were used unless otherwise stated. The precursor and fragment ion mass tolerances were set at 2.0 and 0.9 dalton (Da), respectively. Advanced network options were set as follows: minimum pairs cosine, 0.6; maximum matched fragment ions, 4; and minimum cluster size, 1. For library search, minimum matching peaks was set at 4, with a score threshold of 0.6. The resulting molecular networks were visualized using Cytoscape v3.9.0^71^. Annotated known secondary metabolites by the GNPS spectral library were further assessed by manual evaluation of the matched mass fragments and cross-checking with available published data.

### Genome sequencing and analysis

The genome sequence of BE93 was previously obtained^47^ and deposited in GenBank (www.ncbi.nlm.nih.gov/genbank/) under the accession number JAACZL010000000. The whole genome sequence was visualized using the CGView tool^72^ and functional gene annotations or clusters of orthologous groups (COGs) classifications were generated using the eggNOG-mapper v2^73^. To identify secondary metabolite biosynthetic gene clusters (BGCs), the genome sequence was submitted to antiSMASH 5.0^49^ and PRISM^50^ and analyzed using the default parameters.

### Homology modelling

Homology modelling was employed to obtain the 3D protein model of ivermectin**–**sensitive invertebrate α glutamate-gated chloride channel (GluCl) of *B*. *xylophilus*. Using the *C*. *elegans* protein sequence, a basic local alignment search tool (BLAST) analysis against the genome sequence of *B*. *xylophilus* in the database of national center for biotechnology information (NCBI) allowed the retrieval of a homologous protein with an accession code CAD5232901. The α GluCl PDB crystal structure of *C*. *elegans* (3RHW_A) from the research collaboratory for structural bioinformatics protein data bank (RCSB PDB; https://www.rcsb.org/) was used as the template for the homology modelling through SWISS-MODEL^74^. Quality evaluation of the model was employed through PROCHECK^75^, and ERRAT^76^ at the SAVES server (https://saves.mbi.ucla.edu/). Energy minimization of the model was carried out through steps of steepest descent and conjugate gradient using GROMOS^77^ in the Swiss-PDB viewer environment^78^.

### Molecular docking

Molecular docking was performed using the AutoDock Vina^51^ within the UCSF Chimera^52^ working space. The modelled 3D protein α GluCl of *B*. *xylophilus* and the α GluCl of *C*. *elegans* (3RHW_A) were used as receptors. Chains A and B of the protein channel were selected for the docking experiment, representing the principal (+) subunit and the adjacent complementary (‒) subunit of the channel. Koranimine and ivermectin structures were retrieved from PubChem (https://pubchem.ncbi.nlm.nih.gov/) and RCSB PDB, respectively. The receptors and ligands were prepared using the Dock prep tool in UCSF Chimera with the default parameters. Although the amino acid residues comprising the allosteric site of the receptor are known, blind docking was still performed to investigate the binding potential of koranimine. The molecular docking was carried out with the default parameters of the AutoDock Vina. The resulting receptor-ligand complex configurations and interactions were visualized and analyzed using the BIOVA discovery studio visualizer (https://discover.3ds.com/discovery-studio-visualizer-download). 

## Supplementary Information

Supplementary Information.

## Data Availability

All data generated or analyzed during this study are included in this article and in its supporting information.

## References

[1] Cheng, X. Y., Cheng, F. X., Xu, R. M. & Xie, B. Y. Genetic variation in the invasive process of Bursaphelenchus xylophilus (Aphelenchida: Aphelenchoididae) and its possible spread routes in China. *Hered.* **100**, 356–365 (2007). 10.1038/sj.hdy.6801082 18091770

[2] Mamiya Y (1988). History of pine wilt disease in Japan. J. Nematol..

[3] Futai K (2013). Pine wood nematode, bursaphelenchus xylophilus. Annu. Rev. Phytopathol..

[4] Kuroda, K. Physiological incidences related to symptom development and wilting mechanism. *Pine Wilt Dis.* 204–222 (2008). 10.1007/978-4-431-75655-2_21.

[5] Jones JT, Moens M, Mota M, Li H, Kikuchi T (2008). Bursaphelenchus xylophilus: Opportunities in comparative genomics and molecular host–parasite interactions. Mol. Plant Pathol..

[6] Proença DN, Grass G, Morais PV (2017). Understanding pine wilt disease: Roles of the pine endophytic bacteria and of the bacteria carried by the disease-causing pinewood nematode. Microbiologyopen.

[7] Evans HF, McNamara DG, Braasch H, Chadoeuf J, Magnusson C (1996). Pest risk analysis (PRA) for the territories of the European Union (as PRA area) on Bursaphelenchus xylophilus and its vectors in the genus Monochamus. EPPO Bull..

[8] Mota MM (1999). First report of Bursaphelenchus xylophilus in Portugal and in Europe. Nematology.

[9] Dwinell LD (1997). The Pinewood nematode: Regulation and mitigation. Annu. Rev. Phytopathol..

[10] Takai K, Soejima T, Suzuki T, Kawazu K (2001). Development of a water-soluble preparation of emamectin benzoate and its preventative effect against the wilting of pot-grown pine trees inoculated with the pine wood nematode, bursaphelenchus xylophilus. Pest. Manag. Sci..

[11] Kwon TS, Shin JH, Lim JH, Kim YK, Lee EJ (2011). Management of pine wilt disease in Korea through preventative silvicultural control. For. Ecol. Manage..

[12] Aktar W, Sengupta D, Chowdhury A (2009). Impact of pesticides use in agriculture: Their benefits and hazards. Interdiscip. Toxicol..

[13] Gopal RM, Pomroy WE, West DM (1999). Resistance of field isolates of Trichostrongylus colubriformis and Ostertagia circumcincta to ivermectin. Int. J. Parasitol..

[14] Li JWH, Vederas JC (2009). Drug discovery and natural products: End of an era or an endless frontier?. Science.

[15] Shuikan, A. M. *et al.* Enhancement and identification of microbial secondary metabolites. *Extrem. Microbes Metab. Divers. Bioprospecting Biotechnol. Appl.* (2020). 10.5772/INTECHOPEN.93489.

[16] Fisher MH (1990). Recent advances in avermectin research. Pure Appl. Chem..

[17] Huang, D. *et al.* Identification and characterization of nematicidal volatile organic compounds from deep-sea Virgibacillus dokdonensis MCCC 1A00493. *Molecules* **25**, (2020). 10.3390/molecules25030744 PMC7037310 32050419

[18] Zhai, Y. *et al.* Cyclo(l-Pro–l-Leu) of Pseudomonas putida MCCC 1A00316 isolated from antarctic soil: Identification and characterization of activity against Meloidogyne incognita. *Mol. ***24**, 768 (2019). 10.3390/molecules24040768 PMC6412658 30791605

[19] Kang, M. K. *et al.* Nematicidal activity of teleocidin B4 isolated from Streptomyces sp. against pine wood nematode, Bursaphelenchus xylophilus. *Pest Manag. Sci.* **77**, 1607–1615 (2021). 10.1002/ps.6095 32954637

[20] Liu MJ (2019). Screening, isolation and evaluation of a nematicidal compound from actinomycetes against the pine wood nematode Bursaphelenchus xylophilus. Pest Manag. Sci..

[21] Burg RW (1979). Avermectins, new family of potent anthelmintic agents: Producing organism and fermentation. Antimicrob. Agents Chemother..

[22] Lynagh T, Lynch JW (2012). Ivermectin binding sites in human and invertebrate Cys-loop receptors. Trends Pharmacol. Sci..

[23] Ikeda T (2003). Pharmacological effects of ivermectin, an antiparasitic agent for intestinal strongyloidiasis: Its mode of action and clinical efficacy. Nihon Yakurigaku Zasshi..

[24] Kass IS, Wang CC, Walrond JP, Stretton AOW (1980). Avermectin B1a, a paralyzing anthelmintic that affects interneurons and inhibitory motoneurons in Ascaris. Proc. Natl. Acad. Sci. U. S. A..

[25] Fritz LC, Wang CC, Gorio A (1979). Avermectin B1a irreversibly blocks postsynaptic potentials at the lobster neuromuscular junction by reducing muscle membrane resistance. Proc. Natl. Acad. Sci. U. S. A..

[26] Senges CHR (2018). The secreted metabolome of Streptomyces chartreusis and implications for bacterial chemistry. Proc. Natl. Acad. Sci. U. S. A..

[27] Pye CR, Bertin MJ, Lokey RS, Gerwick WH, Linington RG (2017). Retrospective analysis of natural products provides insights for future discovery trends. Proc. Natl. Acad. Sci. U. S. A..

[28] Ziemert, N., Weber, T. & Medema, M. H. Genome mining approaches to bacterial natural product discovery. *Compr. Nat. Prod. III* 19–33 (2020). 10.1016/B978-0-12-409547-2.14627-X.

[29] Tangerina, M. M. P. *et al.* Metabolomic study of marine Streptomyces sp.: Secondary metabolites and the production of potential anticancer compounds. *PLoS One* **15**, e0244385 (2020). 10.1371/journal.pone.0244385 PMC7751980 33347500

[30] Bedair M, Sumner LW (2008). Current and emerging mass-spectrometry technologies for metabolomics. TrAC Trends Anal. Chem..

[31] Watrous J (2012). Mass spectral molecular networking of living microbial colonies. Proc. Natl. Acad. Sci. U. S. A..

[32] Aron AT (2020). Reproducible molecular networking of untargeted mass spectrometry data using GNPS. Nat. Protoc..

[33] Naman CB (2017). Integrating Molecular Networking and Biological Assays To Target the Isolation of a Cytotoxic Cyclic Octapeptide, Samoamide A, from an American Samoan Marine Cyanobacterium. J. Nat. Prod..

[34] Chao R (2021). Targeted isolation of asperheptatides from a coral-derived fungus using LC-MS/MS-based molecular networking and antitubercular activities of modified cinnamate derivatives. J. Nat. Prod..

[35] Liu J, Nothias LF, Dorrestein PC, Tahlan K, Bignell DRD (2021). Genomic and metabolomic analysis of the potato common scab pathogen Streptomyces scabiei. ACS Omega.

[36] Wang, X., Subko, K., Kildgaard, S., Frisvad, J. C. & Larsen, T. O. Mass spectrometry-based network analysis reveals new insights into the chemodiversity of 28 species in Aspergillus section Flavi. *Front. Fungal Biol.* **0**, 32 (2021). 10.3389/ffunb.2021.719420 PMC10512371 37744124

[37] Shaikh AA, Nothias LF, Srivastava SK, Dorrestein PC, Tahlan K (2021). Specialized metabolites from ribosome engineered strains of streptomyces clavuligerus. Metabolites.

[38] Le, V. T. *et al.* Untargeted metabolomics approach for the discovery of environment-related pyran-2-ones chemodiversity in a marine-sourced penicillium restrictum. *Mar. Drugs* **19**, 378 (2021). 10.3390/md19070378 PMC8305465 34210084

[39] Hartshorn MJ (2007). Diverse, high-quality test set for the validation of protein-ligand docking performance. J. Med. Chem..

[40] Stark JL, Powers R (2012). Application of NMR and molecular docking in structure-based drug discovery. Top. Curr. Chem..

[41] Kitchen, D. B., Decornez, H., Furr, J. R. & Bajorath, J. Docking and scoring in virtual screening for drug discovery: methods and applications. *Nat. Rev. Drug Discov. ***3**, 935–949 (2004). 10.1038/nrd1549 15520816

[42] Cools F (2020). In vitro and in vivo evaluation of in silico predicted pneumococcal UDPG:PP inhibitors. Front. Microbiol..

[43] Bhattacharyya A (2020). Mechanistic insight into the antifungal effects of a fatty acid derivative against drug-resistant fungal infections. Front. Microbiol..

[44] Bharadwaj, S. *et al.* Discovery of Ganoderma lucidum triterpenoids as potential inhibitors against Dengue virus NS2B-NS3 protease. *Sci. Rep.* **9**, 1–12 (2019). 10.1038/s41598-019-55723-5 PMC6911040 31836806

[45] Ferraro, F. *et al.* Cathepsin L Inhibitors with activity against the liver fluke identified from a focus library of quinoxaline 1,4-di-N-oxide derivatives. *Molecules ***24**, 2348 (2019). 10.3390/molecules24132348 PMC6651555 31247891

[46] Ponpandian, L. N. *et al.* Phylogenetic characterization of bacterial endophytes from four Pinus species and their nematicidal activity against the pine wood nematode. *Sci. Rep.* **9**, 1–11 (2019). 10.1038/s41598-019-48745-6 PMC6713757 31462655

[47] Montecillo, J. A. V. & Bae, H. Reclassification of Brevibacterium frigoritolerans as Peribacillus frigoritolerans comb. nov. based on phylogenomics and multiple molecular synapomorphies. *Int. J. Syst. Evol. Microbiol.* **72**, 005389 (2022). 10.1099/ijsem.0.005389 35604831

[48] Nothias, L. F. *et al.* Feature-based molecular networking in the GNPS analysis environment. *Nat. Methods ***17**, 905–908 (2020). 10.1038/s41592-020-0933-6 PMC7885687 32839597

[49] improving cluster detection and comparison capabilities (2021). Blin, K. *et al.* antiSMASH 6.0. Nucleic Acids Res..

[50] Skinnider, M. A. *et al.* Comprehensive prediction of secondary metabolite structure and biological activity from microbial genome sequences. *Nat. Commun. ***11**, 1–9 (2020). 10.1038/s41467-020-19986-1 PMC7699628 33247171

[51] Trott O, Olson AJ (2010). AutoDock Vina: Improving the speed and accuracy of docking with a new scoring function, efficient optimization, and multithreading. J. Comput. Chem..

[52] Pettersen EF (2004). UCSF Chimera—a visualization system for exploratory research and analysis. J. Comput. Chem..

[53] Evans BS, Ntai I, Chen Y, Robinson SJ, Kelleher NL (2011). Proteomics-based discovery of koranimine, a cyclic imine natural product. J. Am. Chem. Soc..

[54] Hibbs, R. E. & Gouaux, E. Principles of activation and permeation in an anion-selective Cys-loop receptor. *Nat. ***474**, 54–60 (2011). 10.1038/nature10139 PMC3160419 21572436

[55] Del Boccio P (2003). Liquid chromatography–tandem mass spectrometry analysis of oleuropein and its metabolite hydroxytyrosol in rat plasma and urine after oral administration. J. Chromatogr. B.

[56] Du L, Sánchez C, Chen M, Edwards DJ, Shen B (2000). The biosynthetic gene cluster for the antitumor drug bleomycin from Streptomyces verticillus ATCC15003 supporting functional interactions between nonribosomal peptide synthetases and a polyketide synthase. Chem. Biol..

[57] Weber, G., Schörgendorfer, K., Schneider-Scherzer, E. & Leitner, E. The peptide synthetase catalyzing cyclosporine production in Tolypocladium niveum is encoded by a giant 45.8-kilobase open reading frame. *Curr. Genet. ***26**, 120–125 (1994). 10.1007/BF00313798 8001164

[58] Walton JD (2006). HC-toxin. Phytochemistry.

[59] Xu Y (2008). Biosynthesis of the cyclooligomer depsipeptide beauvericin, a virulence factor of the entomopathogenic Fungus Beauveria bassiana. Chem. Biol..

[60] Pan R, Bai X, Chen J, Zhang H, Wang H (2019). Exploring structural diversity of microbe secondary metabolites using OSMAC strategy: A literature review. Front. Microbiol..

[61] Seyedsayamdost MR (2019). Toward a global picture of bacterial secondary metabolism. J. Ind. Microbiol. Biotechnol..

[62] Sharma OP (2012). Modeling, docking, simulation, and inhibitory activity of the benzimidazole analogue against β-tubulin protein from Brugia malayi for treating lymphatic filariasis. Med. Chem. Res..

[63] Becker JE, Moore RE, Moore BS (2004). Cloning, sequencing, and biochemical characterization of the nostocyclopeptide biosynthetic gene cluster: molecular basis for imine macrocyclization. Gene.

[64] Krunic A (2010). Scytonemides A and B, cyclic peptides with 20S proteasome inhibitory activity from the cultured cyanobacterium scytonema hofmanii. J. Nat. Prod..

[65] Zipperer, A. *et al.* Human commensals producing a novel antibiotic impair pathogen colonization. *Nat. ***535**, 511–516 (2016). 10.1038/nature18634 27466123

[66] Otero A, Chapela M-J, Atanassova M, Vieites JM, Cabado AG (2011). Cyclic Imines: Chemistry and mechanism of action: a review. Chem. Res. Toxicol.

[67] Peachey LE (2017). P-glycoproteins play a role in ivermectin resistance in cyathostomins. Int. J. Parasitol. Drugs Drug Resist..

[68] Prichard, R. K. Ivermectin resistance and overview of the Consortium for Anthelmintic Resistance SNPs. *Expert Opin. Drug Discov.* **2**, (2007). 10.1517/17460441.2.S1.S41 23489032

[69] Chambers, M. C. *et al.* A cross-platform toolkit for mass spectrometry and proteomics. *Nat. Biotechnol. ***30**, 918–920 (2012). 10.1038/nbt.2377 PMC3471674 23051804

[70] Pluskal T, Castillo S, Villar-Briones A, Orešič M (2010). MZmine 2: Modular framework for processing, visualizing, and analyzing mass spectrometry-based molecular profile data. BMC Bioinf..

[71] Shannon P (2003). Cytoscape: A software environment for integrated models of biomolecular interaction networks. Genome Res..

[72] Stothard P, Wishart DS (2005). Circular genome visualization and exploration using CGView. Bioinformatics.

[73] Cantalapiedra, C. P., Hern Andez-Plaza, A., Letunic, I., Bork, P. & Huerta-Cepas, J. eggNOG-mapper v2: Functional annotation, orthology assignments, and domain prediction at the metagenomic scale. *Mol. Biol. Evol.* **38**, 5825–5829 (2021). 10.1093/molbev/msab293 PMC8662613 34597405

[74] Waterhouse A (2018). SWISS-MODEL: Homology modelling of protein structures and complexes. Nucleic Acids Res..

[75] Laskowski, R. A., MacArthur, M. W., Moss, D. S., Thornton, J. M. & IUCr. PROCHECK: a program to check the stereochemical quality of protein structures. *urn:issn:0021–8898* **26**, 283–291 (1993).

[76] Colovos C, Yeates TO (1993). Verification of protein structures: Patterns of nonbonded atomic interactions. Protein Sci..

[77] Scott WRP (1999). The GROMOS biomolecular simulation program package. J. Phys. Chem. A.

[78] Kaplan W, Littlejohn TG (2001). Swiss-PDB Viewer (Deep View). Brief. Bioinform..

