# Supplementary material for: In silico analysis of koranimine, a cyclic imine compound from Peribacillus frigoritolerans reveals potential nematicidal activity
Source: Sci Rep. 2022 Nov 7;12:18883. doi: 10.1038/s41598-022-20461-8 (PMC9640594; doi:10.1038/s41598-022-20461-8)
Supplement: Supplementary file 1 — Supplementary Information. [file 41598_2022_20461_MOESM1_ESM.pdf]

Supplementary Figures and Tables

Supplementary Table 1. Processed table from the LC-MS/MS data analyzed using MZmine 2.

row ID*	row m/z	row retention time	BE93_R2A.mzML Peak area	BE93_NB.mzML Peak area	BE93_LB.mzML Peak area	BE93_TSB.mzML Peak area
2	197.0541697	14	5.05E+08	3.31E+08	9.58E+08	4.83E+08
3	211.1000061	18	1.22E+09	1.41E+09	2.28E+09	2.64E+09
5	261.0514594	13	6.57E+08	2.44E+08	5.10E+08	1.92E+09
6	340.2874908	24	2.55E+08	2.66E+08	2.49E+08	2.65E+08
7	371.1000061	25	4.29E+08	7.79E+07	1.01E+08	2.02E+08
8	385.1000061	29	6.22E+08	1.20E+08	2.33E+08	3.94E+08
9	453.25	20	3.35E+08	3.34E+08	3.38E+08	3.36E+08
10	679.3499756	24	2.54E+08	2.61E+08	2.48E+08	2.83E+08
11	235.0479196	3.6	1.65E+08	2.12E+07	4.99E+08	6.84E+08
12	245.0625038	20	3.13E+08	4.05E+08	5.21E+08	2.74E+09
13	243.0077386	24	3.17E+08	1.66E+08	3.65E+08	1.95E+08
14	138	20	6.15E+08	2.70E+08	9.59E+08	1.18E+09
15	347.0499878	22	2.31E+08	2.78E+07	2.97E+08	4.37E+08
16	701.3499756	24	2.13E+08	1.98E+08	2.42E+08	2.43E+08
17	219.0750046	25	2.13E+08	2.01E+07	1.09E+07	4.54E+07
18	279.0624924	13	4.91E+08	8.82E+07	6.92E+08	3.44E+08
19	383.1000061	27	4.11E+08	2.24E+08	1.16E+08	1.96E+08

20	283.0499878	14	2.95E+08	824787	6.81E+08	5.38E+08
21	475.2000122	20	1.76E+08	1.92E+08	2.01E+08	2.55E+08
22	147.0500031	14	4.87E+08		8.23E+08	6.91E+07
23	419.0499878	30	1.76E+08	2.71E+07	7339755	1.67E+07
25	804.3279597	44	1.56E+09	1.59E+08	1.77E+08	3.45E+09
27	440.1535666	43	3.82E+08	3.31E+08	1.75E+07	1.27E+08
28	426.1499939	41	2.13E+08	1.42E+08		
30	401.1499939	29	9.55E+07	9.23E+07	8.33E+07	9.81E+07
31	220.0592623	22	1.21E+08		3.06E+08	2.01E+07
37	343.0624924	17	1.25E+08	3094458	3.19E+07	3.90E+07
38	239	21	6.30E+07	1.39E+08	4.23E+08	3.44E+08
41	229.0355169	15	1.19E+08	9.21E+07	7.15E+08	4.16E+08
42	267.0437393	30	7.43E+07	4459639	6904997	3642243.07
43	227.1000061	13	9.28E+07	1.10E+08	7.77E+07	8.26E+07
44	164.0468779	7.3	7.99E+07	6.94E+07	2.30E+08	1.68E+08
45	298	11	8.43E+07	7.42E+07	6.49E+07	1.98E+08
51	266.0472124	35	6.98E+07		8.61E+07	1873981.63
52	250.0500031	34	9.93E+07	1.01E+08	2.16E+08	5.49E+08
58	282.2187576	54	7.07E+07	1.12E+08	3.76E+07	1.86E+07
60	382.0812492	34	4.92E+07	1.39E+07	4.10E+07	4.33E+07
77	256.2000122	54	6.98E+07	4.29E+07	4.82E+07	5.82E+07

78	213.1000061	22	7.90E+07	1.20E+08	7.49E+08	8.23E+08
80	146.0250015	24	7.15E+07	1.95E+08	9.61E+07	6.28E+07
86	284.0875015	23	3.84E+07	4.18E+08	4.39E+08	4.80E+08
94	280.1985402	52	6.76E+07	7.86E+07	6.59E+07	3.97E+07
96	242.1999969	52	6.47E+07	2.82E+07	1.50E+07	1709740.65
100	227.1000061	26	3.24E+07	5.28E+07	2.01E+08	9.17E+08
102	137	4.7	3.52E+07	6.63E+08	2.18E+08	2.13E+08
105	261.0624924	27	3.06E+07	8.59E+07	3.86E+08	9.20E+08
120	169.0125008	15	8.17E+07	6.76E+07	1.78E+08	1.99E+08
125	185.0708373	15	3.22E+07	3.75E+07	1.05E+08	4.61E+08
129	169.0500031	8.5	3.00E+07	6.42E+07	3.77E+08	4.51E+08
135	110.0874996	3.6	6279400	1.44E+08	1.07E+08	5.84E+08
139	340.1000061	27	1.30E+07		1.40E+08	1.73E+08
142	166.0125008	8.3	2.36E+07	2.37E+08	4.98E+08	2.56E+08
144	195.0500031	11	2.25E+07	7.66E+07	1.92E+08	1.26E+08
148	171.0553062	12	1.95E+07	1.54E+08	1.23E+08	1.38E+08
151	155.0500031	7	2.34E+07	7.76E+08	7.42E+07	1.40E+08
154	226.9187508	2.9	4084656	1.09E+08	3.10E+07	5935563.94
170	295.0499878	29	1.27E+07	1.85E+07	6.49E+07	3.51E+08
171	229.064586	15	1.98E+07	1.19E+07	3.59E+08	1.57E+09
175	227.0583369	15	1.64E+07	6.82E+08		1673810.66

181	319.2000122	52	2.67E+07	2.56E+07	2.26E+07	2.25E+07
187	199.0500031	7.8	1.59E+07	3.28E+07	9.23E+07	2.34E+08
188	201.05417	9.8	1.43E+07	3804870	2.10E+08	3.00E+08
191	169.0498046	12	1.58E+07	4696578	1.39E+08	2.32E+08
192	120.0625019	8.3	1.45E+07	1.22E+08	1.93E+08	6.33E+07
198	277.1000061	17	1.42E+07	1.71E+07	1.84E+08	3.83E+08
201	275.183936	52	2.18E+07	2.05E+07	2.45E+07	7049855.63
219	387.2000122	52	3.01E+07	7536653	3.75E+07	8563853.26
242	336.2375031	52	1.64E+07	1.46E+07	1.69E+07	1.53E+07
244	306.0499878	23	1.18E+07	1.51E+08	1.79E+08	2.37E+08
274	765.3729261	53	4.38E+07	7.19E+07	1.25E+07	172247.461
277	414.2140675	51	2.09E+07	303989.7	475987	488828.925
322	277.0812492	19	3320808	5027876	4.28E+07	3.02E+08
350	285.0624924	9.9	3607384	3.09E+07	6.33E+07	2.64E+08
380	284.0750008	21	4900168	8.87E+07	2.07E+07	1.57E+08
457	663.2916565	55	1.04E+07	2940666	4887182	110466.492
559	213.1000023	40	878417.5	1071069	7.51E+07	3.09E+08
566	313.0875015	49	4193142	8087895	1.04E+07	3.15E+07
582	251.125	55	5114305	6375429	2043587	979266.614
665	531.3187408	56	2447589	192899.5	478222	2154981.07
672	269.1281261	51	4660973	5046578	1101521	4.77E+07

705	496.3291673	55	2408563	2205758	267373	1301236.4
719	295.1124954	51	1965950	2140326	6114477	2.43E+07
765	148.9749985	55	1275250	1634210	1208511	171465.248
816	391.2000122	59	1715697	547297	1093476	839303.685
870	284.2250061	57	1557755	2353089	1923939	2644702.9
911	391.244165	55	1465034	1299297	984571	332950.241
979	584.3270683	53	403892.9	143668.2	8408356	9661979.71
987	391.2708276	55	812510.6	465231.8	452035	149478.551
1000	309.1573003	55	456356.5	206284.4	1208691	122530.136
1006	663.299998	56	136079.2	1143140	75204.5	93493.0295
1189	422.1750031	37		3.38E+08		2709619.52
1194	369.125	37		2.32E+08		798204.451
1212	147.0895882	2.8		5.72E+07	9651893	6864018.04
1246	146.0250015	12	1.90E+07	4.75E+07	1.48E+08	9.25E+07
1270	261.0493934	13	5.48E+08	4.31E+07	2.11E+09	8.46E+08
1277	181.048753	16	3.33E+07	3.82E+07	5.72E+08	7.15E+08
1312	249.0944485	3.5		8304105	808995	4.42E+07
1402	199.0805605	18	566908.7	6882961	8718170	4.85E+07
1418	231.0416692	19		1.30E+07	3.53E+07	1.40E+08
1619	279.0333252	24		1467297	2.24E+07	1.18E+08
1655	423.1770871	51	231500.9	8338574	320880	355950.234

1770	283.2000122	53	684822.9	9091966	1.85E+07	1.47E+08
1901	515.3229243	59	14505.49	160496.5	165493	4826
1991	393.2474945	55	156825.3	271198.4	458076	4339737.84
2061	251.086463	3.6	1.78E+07	913971.7	1.04E+08	9.83E+07
2062	267.0499878	21			1.87E+08	2.95E+08
2064	203.0033335	12	2267657		1.03E+08	1.86E+08
2069	284.1000061	27			8.02E+07	1.20E+08
2071	822.3020706	42	9561268	4.92E+07	9.60E+08	1.30E+09
2131	276.0725011	13	9084312		1.93E+07	1.66E+08

* Note: The row ID numbers presented here were “raw” and were not changed to numbers 1 to 110 to represent the 110 spectral features.

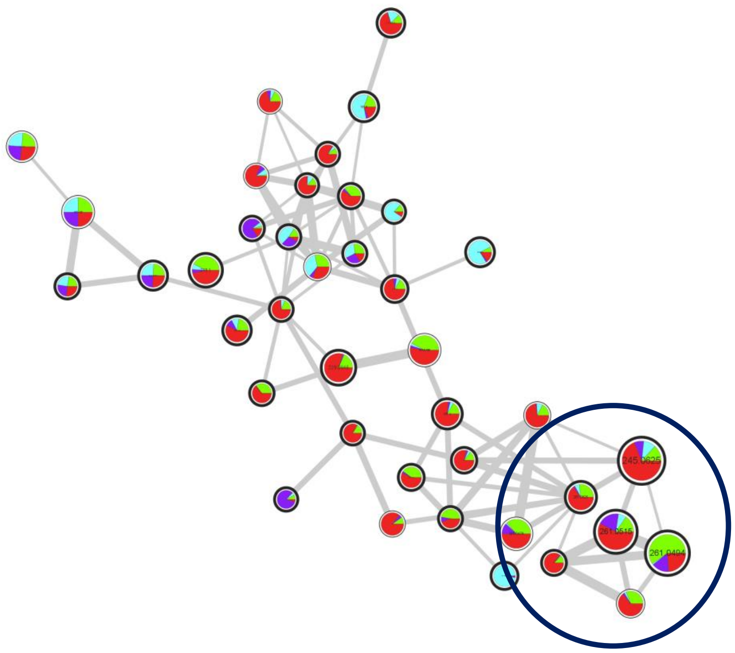

Supplementary Figure 1. Molecular network of ions ranging from 211.10 to 261.05 m/z (circled), representing compounds of the diketopiperizines family.

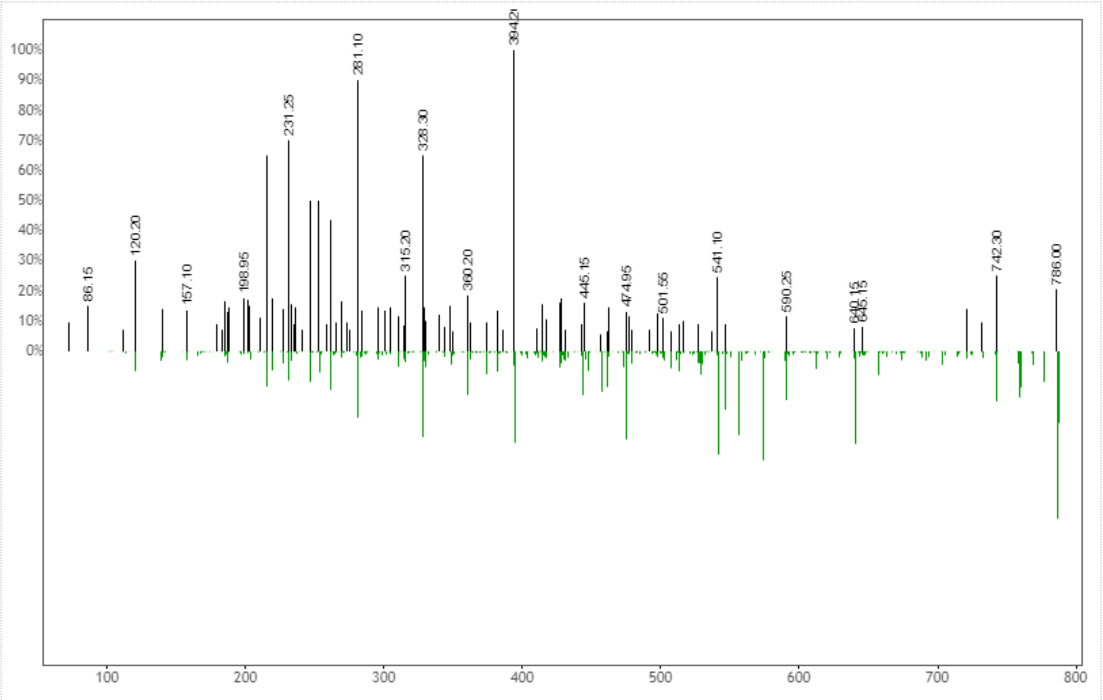

Supplementary Figure 2. MS/MS spectral data of koranimine from the GNPS library (bottom) matching with the MS/MS spectrum of the detected compound in the *P. frigoritolerans* culture extracts (top).

(a)

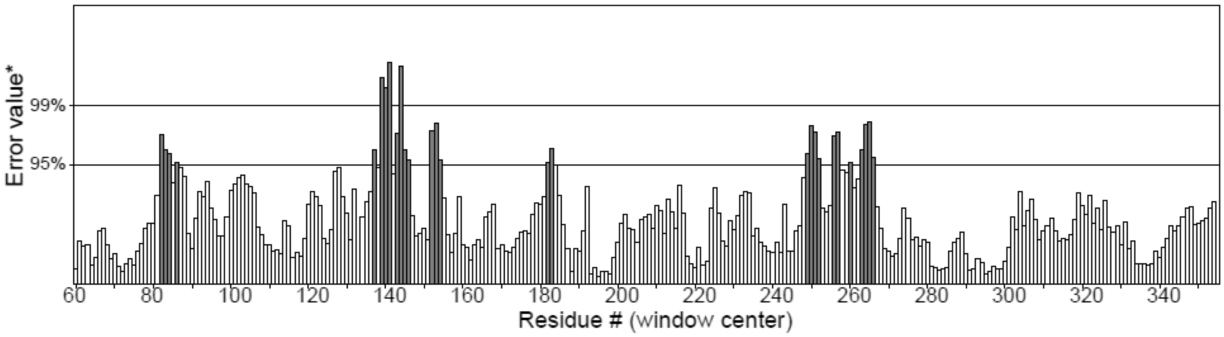

Error value*
99%
95%
60 80 100 120 140 160 180 200 220 240 260 280 300 320 340
Residue # (window center)

(b)

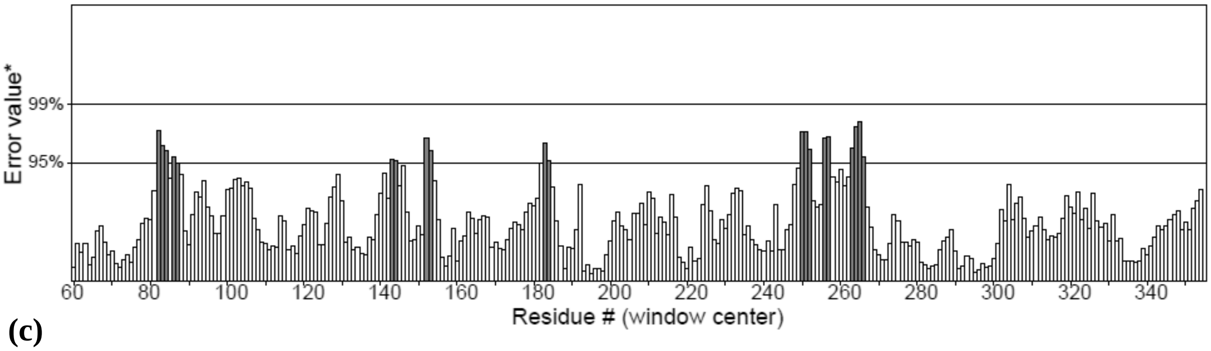

Error value*
99%
95%
60 80 100 120 140 160 180 200 220 240 260 280 300 320 340
Residue # (window center)

(c)

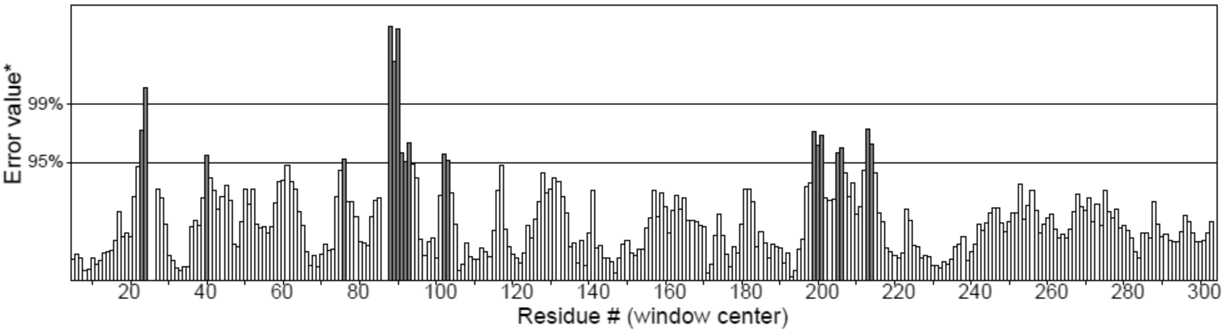

Error value*
99%
95%
20 40 60 80 100 120 140 160 180 200 220 240 260 280 300
Residue # (window center)

(d)

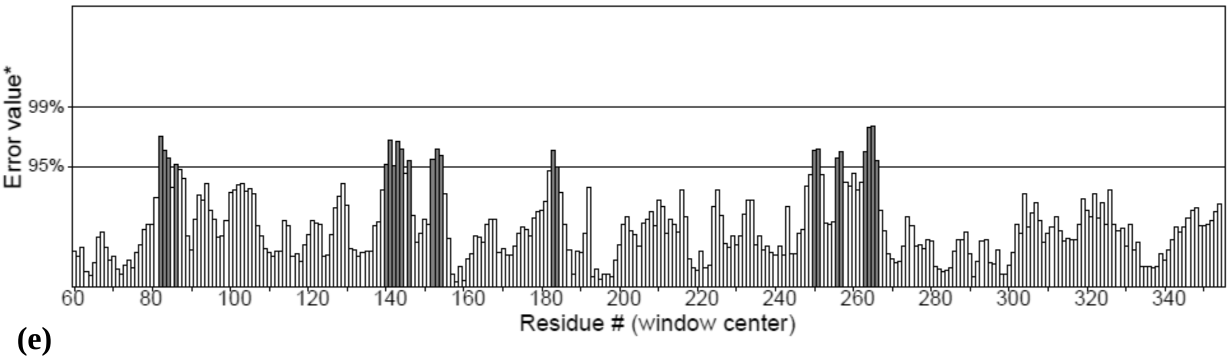

(e)

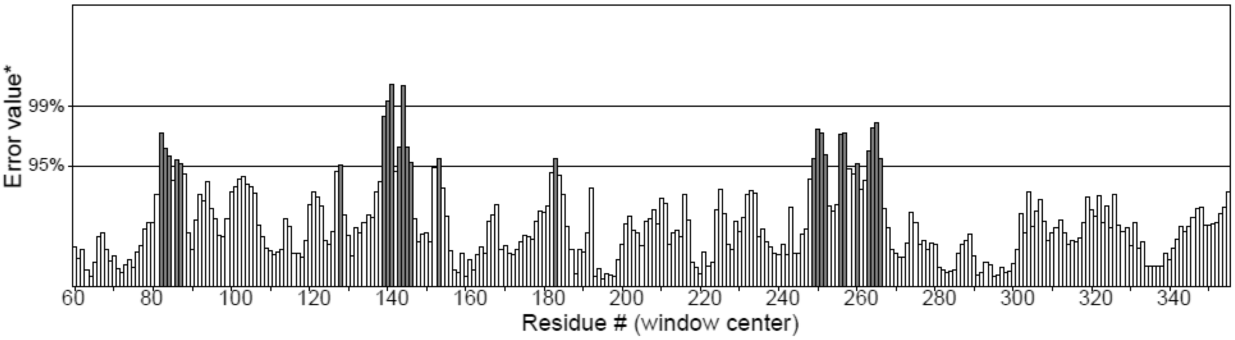

Supplementary Figure 3. ERRAT plot showing that the generated 3D model has a good resolution as the regions of the modelled structure that can be rejected at the 95% and 99% of confidence is very low with overall quality factor of 92.136%. Figures a-e represent each chain of the modelled protein.
